# Evidence of Mars‐Van‐Krevelen Mechanism in the Electrochemical Oxygen Evolution on Ni‐Based Catalysts

**DOI:** 10.1002/anie.202101698

**Published:** 2021-05-26

**Authors:** Jorge Ferreira de Araújo, Fabio Dionigi, Thomas Merzdorf, Hyung‐Suk Oh, Peter Strasser

**Affiliations:** ^1^ Department of Chemistry Chemical Engineering Division Technical University of Berlin Straße des 17. June 124 10623 Berlin Germany; ^2^ Clean Energy Research Center Korea Institute of Science and Technology (KIST) Hwarangro 14-gil 5, Seongbuk-gu 02792 Seoul Republic of Korea

**Keywords:** alkaline OER catalyst, differential electrochemical mass spectrometry, isotope ^18^O, lattice oxygen evolution

## Abstract

Water oxidation is a crucial reaction for renewable energy conversion and storage. Among the alkaline oxygen evolution reaction (OER) catalysts, NiFe based oxyhydroxides show the highest catalytic activity. However, the details of their OER mechanism are still unclear, due to the elusive nature of the OER intermediates. Here, using a novel differential electrochemical mass spectrometry (DEMS) cell interface, we performed isotope‐labelling experiments in ^18^O‐labelled aqueous alkaline electrolyte on Ni(OH)_2_ and NiFe layered double hydroxide nanocatalysts. Our experiments confirm the occurrence of Mars‐van‐Krevelen lattice oxygen evolution reaction mechanism in both catalysts to various degrees, which involves the coupling of oxygen atoms from the catalyst and the electrolyte. The quantitative charge analysis suggests that the participating lattice oxygen atoms belong exclusively to the catalyst surface, confirming DFT computational hypotheses. Also, DEMS data suggest a fundamental correlation between the magnitude of the lattice oxygen mechanism and the faradaic efficiency of oxygen controlled by pseudocapacitive oxidative metal redox charges.

## Graphical abstract

Differential electrochemical mass spectrometry using a hanging droplet cell confirmed the occurrence of Mars‐van‐Krevelen lattice oxygen evolution reaction mechanism in both Ni(OH)_2_ and NiFe layered double hydroxide nanocatalysts. This mechanism involves the coupling of oxygen atoms from the catalyst surface and the electrolyte.

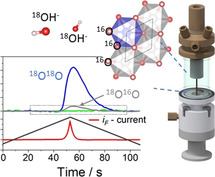

## Introduction

Future large‐scale reductive electrochemical generation of fuels and chemicals at electrolyzer cathodes will require reactions and catalysts at the counter anode that facilitate the release of protons (H^+^) and electrons (e^−^) with maximum efficiency. The electrocatalytic oxygen evolution reaction (OER) or electrochemical water oxidation reaction leading to the formation of O_2_, electrons, and protons is such a key counter reaction. The OER catalysts activity is assumed to be kinetically controlled by surface binding energies between catalytic active surface sites and reactive oxygenated intermediate species.^[1]^ A molecular understanding of energy‐ and cost efficient catalysts for the OER is vital for the design of advanced anodes for water electrolyzers[^2^, ^3^] or CO_2_‐water co‐electrolyzers for the generation of hydrogen or carbonaceous products, respectively.[^4^, ^5^]

Ni‐based materials are among the most active and energy‐efficient OER catalysts for alkaline electrolyzer anodes. The catalytic active state of Ni‐based catalysts is their γ‐phase that forms from the inactive α‐ or β‐phase. The activity of γ‐NiOOH was shown to increase drastically after the addition of Fe under anodic conditions^[6]^ with reported optimal molar Fe:Ni ratios ranging between 0.1 and 0.5.^[7]^ The catalytic active γ‐NiFe OER catalyst presents a layered crystalline structure with intercalated cations between brucite‐type metal oxide layers, in which the metal atoms occupy the center of edge‐connected octahedra. In contrast, the non‐active α‐phase is characterized by intercalated anions, and it is referred to as α‐ NiFe layered double hydroxide (LDH). By structural analogy with γ‐NiOOH, the OER active deprotonated phase that forms by oxidation of α‐NiFe LDH is referred to as γ‐NiFe LDH. Over the years, distinct hypotheses about the nature of the catalytically active site on the brucite layers were put forward.[^1b^, ^8^] Earlier hypotheses include single Fe active sites^[8c]^ or single Ni sites.^[9]^ Later, there emerged heightened interest in the mechanistic role of the oxygen ligands of the edge‐connected M‐O octahedra of the 2D brucite layers.^[10]^

Recently, a new model of the bulk structure of the catalytically active γ‐NiFe LDH phase and of its active surface sites was brought to the fore.^[1b]^ What distinguished this latest model from earlier ones was the primary catalytic role of specific μ_2_‐oxygen bridges between neighboring Ni and Fe sites, while explicitly accounting for i) a reversible cation and water intercalation in the interlayer space and ii) non‐covalent interactions between the interlayer species and the Ni/FeOx brucite layers.^[1b]^ The reactivity of this structural and mechanistic model relied on a direct involvement of surface lattice oxygen ligands in the elementary catalytic reaction mechanisms, also referred to as a surface lattice oxygen evolution reaction (LOER) mechanism. For NiFe LDH, a particular surface LOER mechanism has been proposed where, following a Mars‐van‐Krevelen‐type mechanism, the surface lattice oxygen atom combines with oxygen from the electrolyte forming molecular oxygen and leaving a vacancy, which is then filled by a hydroxide ion from the electrolyte.^[1b]^ The LOER character of state‐of‐art NiFe LDH anodes of alkaline water electrolyzers has remained a contentious issue and calls for experimental verification or dismissal using atomic‐level analytics.

To gain atomic‐level experimental insights into operating NiFe‐based OER catalysts, most earlier studies employed a combination of in situ/*operando* X‐ray absorption spectroscopy (XAS)[^8c^, ^11^] Mössbauer spectroscopy,^[12]^ and voltammetric techniques. However, this set of techniques is not suitable to discriminate whether or not a LOER is present.^[13]^ To achieve this, Differential Electrochemical Mass Spectrometry (DEMS), where product sampling occurs directly from the electrified liquid‐solid interface is most suitable. Earlier DEMS studies on a bimetallic NiFe mass‐selected nanoparticle model catalyst^[14]^ as well as a solvothermally prepared NiFe LDH OER catalyst revealed important details on the stability, activity, and the faradaic efficiency.[^11a^, ^15^] In almost all previous DEMS studies, the electrochemical flow cell architecture involved a mono‐ or a dual thin‐electrolyte layer type. In these DEMS cells, volatile products generated inside a very thin electrolyte film between electrode and a hydrophobic membrane transition into the differentially pumped vacuum system for detection. Thin layer cells suffer from severe mass transport limitations and don't sustain large catalytic current densities. Moreover, they require large electrolyte volumes, which is problematic for studies with expensive isotope labelled electrolytes available only at micro‐ or milliliter scale. This is why innovative bulk electrolyte DEMS cells with small‐volume designs are needed.

In this contribution, we use a novel DEMS cell interface to experimentally test a recently reported computational hypothesis regarding the participation of surface lattice oxygen ligands, which is a LOER, in the OER catalysis on α‐/γ‐NiFe LDH catalysts. Isotope labelling results indeed suggest the presence of a LOER for both liquid precursor‐derived γ‐NiFe LDH catalysts as well as a Fe‐free β‐/γ‐NiO_*x*_H_*y*_ reference catalyst. Crystalline Ir reference oxides showed no LOER. We further unravel and discuss a previously overlooked correlation between the faradaic efficiency and the contribution of the LOER for various catalyst systems. This relation calls for chemical or synthetic measures to minimize the LOER character of OER catalysts in order to maintain high faradaic efficiency. Finally, we demonstrate the ability of the present time‐resolved DEMS technique to accurately deconvolute *faradaic charge* stored in molecular oxygen from *pseudocapacitive charge* stored in the catalyst surface. This previously inaccessible charge balance analysis quantifies the anodic Ni^3+^ and Ni^4+^redox charges as a function of applied electrode potential under catalytic operating conditions.

## Results and Discussion

### Differential Electrochemical Mass Spectrometry in a Hanging Droplet Cell

**Figure 1:**
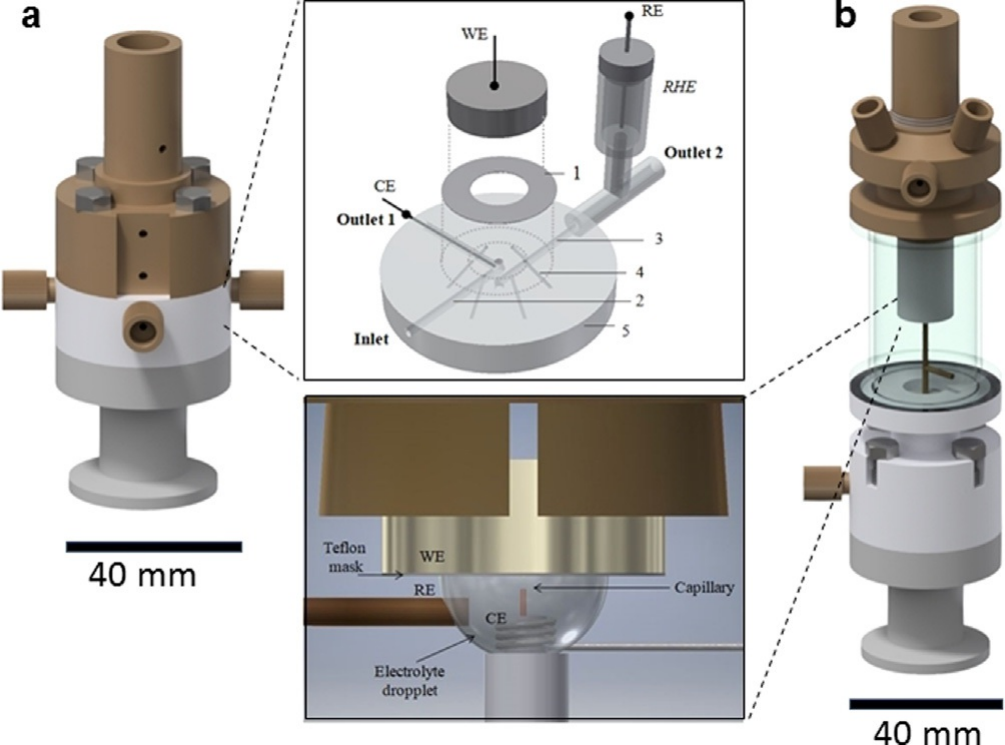
Electrochemical flow cells for differential electrochemical mass spectrometry (DEMS) setup. a) the DEMS dual thin‐layer electrolyte flow cell with close up of main components: working electrode, WE, reversible hydrogen electrode (RHE) as reference electrode, RE, and the Pt counter electrode, CE; 1) Teflon gasket between each cell stack, 2) the inlet channel, 3) the outlet channel, 4) the internal channel and 5) the bulk flow channel stack. b) Illustration of the DEMS hanging droplet cell design showing the actual electrolyte droplet and the main components working electrode (WE) and Teflon mask, the glass capillary, the Ag/AgCl reference electrode, RE, and the Pt counter electrode, CE.

A new differential electrochemical mass spectrometry (DEMS) setup (LIQUIDLOOP GmbH) (Figure S1) was used in this work employing two distinct electrochemical liquid/vacuum cell interfaces (Figure 1 and Figure S2). The dual thin‐layer electrolyte cell (“thin layer cell”) design is based on earlier similar approaches.^[16]^ It is a robust and reliable interface design that requires relatively large electrolyte volumes. It consists of two horizontal parallel disk‐shaped compartments with connecting liquid channels (Figure 1 a). Computational details about the flow conditions and shear stress distributions inside the dual thin‐layer electrolyte flow cell are provided in the Supporting Information and Figure S3.

The “hanging droplet DEMS flow cell” (Figure 1 b) is a new design (see Figure S2 and S4). It was developed for isotope labelling experiments because it allows experiments with microliter scale electrolyte volumes (typically 20–50 μL). This capability is useful whenever expensive solvents or electrolytes are, for instance, isotope‐labelled compounds. The electrochemical measurements are performed inside a hanging electrolyte droplet, the volume of which is maintained under constant in/out flow conditions. The outlet flow ranged typically at 1 μL s^−1^. The inlet tube was placed at 2 mm from the electrode. Reaction products were withdrawn through a capillary placed at 500 μm distance from electrode. The capillary is a concentric tube inside the inlet flow tube. The reaction products are collected together with the electrolyte solution and introduced into a disk‐shaped compartment where a PTFE membrane acts as the interface between the liquid and vacuum. A Ag/AgCl electrode served as reference electrode.

### Mass Spectrometric and Faradaic Voltammetry in Non‐Labelled Conditions

The OER catalysts addressed here comprised a α‐/γ‐NiFe LDH and a β‐Ni(OH)_2_/ γ‐NiO(OH)_*x*_ powder thin film catalysts and were compared to a crystalline Ir oxide electrocatalyst.

**Figure 2:**
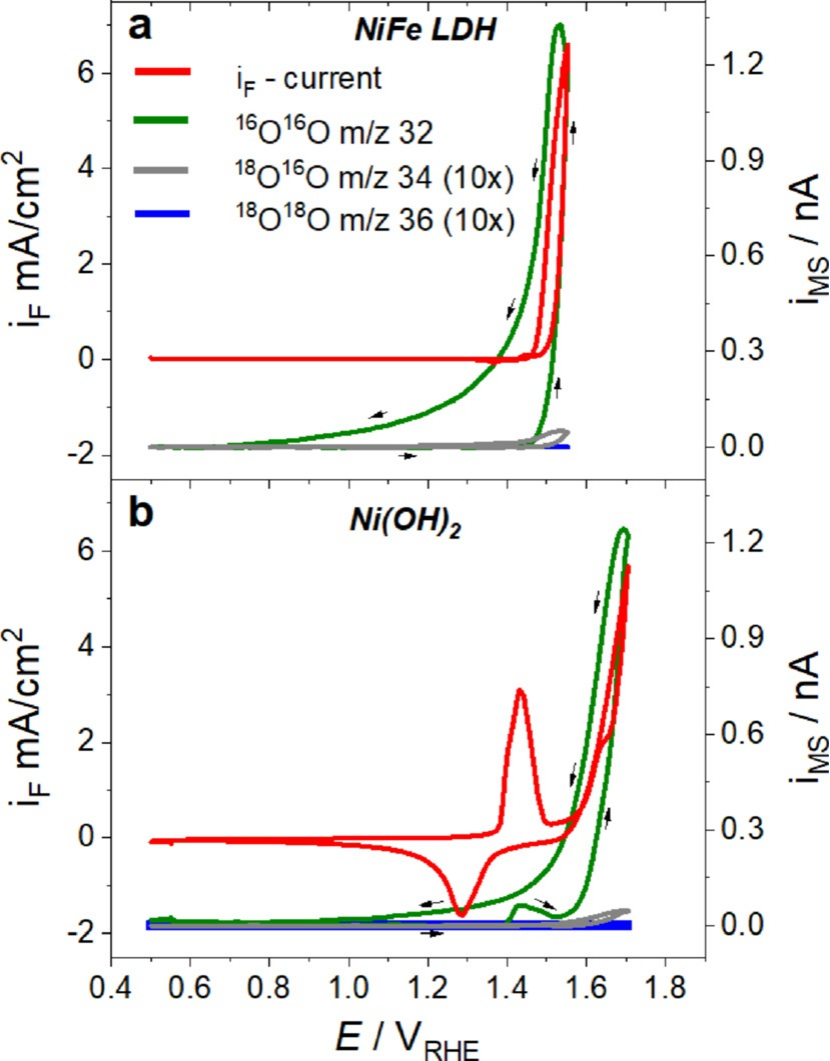
Mass spectrometric cyclic voltammograms (MSCVs), i_MS_, of a) the NiFe LDH and b) the β‐Ni(OH)_2_ OER electrocatalyst. The data was obtained in the dual thin‐layer DEMS flow cell setup in 0.1 M K^16^OH at a scan rate 5 mV s^−1^ up to current density around 6 mA cm^−2^. Plotted are signals of *m*/*z*=32 of ^16^O^16^O (green line), and *m*/*z*=34 and 36 corresponding to the naturally abundant isotopes ^16^O^18^O (grey line, intensity ×10) and ^18^O^18^O (blue line, intensity ×10).

At the outset of this study, the DEMS thin film cell was used to characterize the surface voltammetry and O_2_ faradaic efficiency (“FE_O2_”) of the catalysts in non‐isotope‐enriched (non‐labelled) environments with natural ^16^O/^18^O abundances. After a cyclic voltammetric (CV) activation protocol, faradic voltammograms and simultaneous mass spectrometric cyclic voltammograms (MSCV) were recorded between +0.5 V_RHE_ and the point when 6 mA cm^−2^ was reached at a scan rate of 5 mV s^−1^ (Figure 2). MSCVs tracked the three mass currents of *m*/*z*=32 of ^16^O_2_ (^16^O^16^O) (green line), *m*/*z*=34 of ^16^O^18^O (grey line), and *m*/*z*=36 of ^18^O_2_ (blue line in Figure 2).

The faradaic CVs of the NiFe LDH and Ni(OH)_2_ catalysts are plotted together with their associated MSCVs in Figure 2 a and b. The faradaic CVs obtained in the DEMS cell were nearly identical in shape to those reported previously in conventional three electrode cell and electrode setups, which validates the cell and electrode design.^[17]^ In the anodic scan direction, the MSCVs closely traced the CVs, while on the cathodic scans mass currents revealed characteristic tailings. Such tailings may originate from slow diffusional O_2_ transport out of/across the porous catalyst film.[^11a^, ^18^] However, we observed some tailing in non‐porous thin Ni oxide layers, as well (see Supporting Note 1), implying that the origin of the tailings might be at least in part related to the charge (hole) storage mechanism and slow discharge in Ni‐based OER catalysts. No other volatile products than oxygen (e.g. CO_2_ at *m*/*z* 44) were detected.

For the Ni(OH)_2_ catalyst, the *m*/*z*=32 MSCV featured an unexpected quite cathodic (“low”) onset potential of ^16^O_2_ formation near +1.41 V_RHE_ (green line in Figure 2 b) tracing closely the well‐documented Ni(II+)(OH)_2_ → Ni(III+)OOH redox wave (red line). This ^16^O_2_ generation, however, appears transient in nature, which points to an incomplete reduction of Ni(III+)OOH to Ni(II+)(OH)_2_ during an earlier cathodic scan resulting in oxidative charge trapped by the formation of poorly conductive Ni(OH)_2_ domains.^[19]^ Once the electrode potential was swept anodically again, the Ni(OH)_2_ → NiOOH oxidation re‐occurred restoring a conductive catalyst layer. As a result of this, the trapped hole charges could now discharge by reacting with the electrolyte molecules, resulting in the transient evolution of molecular oxygen.

In bimetallic NiFe LDH catalysts, the partial overlap of the anodic Ni(OH)_2_ → NiOOH wave with the voltammetric OER onset makes an accurate estimate of the onset potential of sustained O_2_ evolution during potential sweep measurements difficult. MSCVs, however, are able to provide them accurately. Figure 2 a shows that the ^16^O_2_ evolution onset of the NiFe LDH catalyst occurred at +1.47 V_RHE,_ hence slightly more cathodic compared to Ni(OH)_2_ (+1.55 V_RHE_ ) (Figure 2 b).

In Figure 2, the mean O_2_ faradic efficiency, FE_O2_, of NiFe LDH (obtained by integrating the MSCV from and back to +0.5 V_RHE_, while integrating the anodic faradaic current only) was 90 %, while that of Ni(OH)_2_ was 62 % (see details in Supporting Note 2).

The signals of the other two oxygen isotopes (^16^O^18^O and ^18^O_2_) were at least 2 orders of magnitude smaller than that of ^16^O_2_. While the ^16^O^18^O signal displayed a weak rise at the most anodic potentials, the signal of ^18^O_2_ was too low to discern a detailed current/potential response.

### ^18^O Isotope‐Enriched Mass Spectrometric and Faradaic Voltammetry

**Figure 3:**
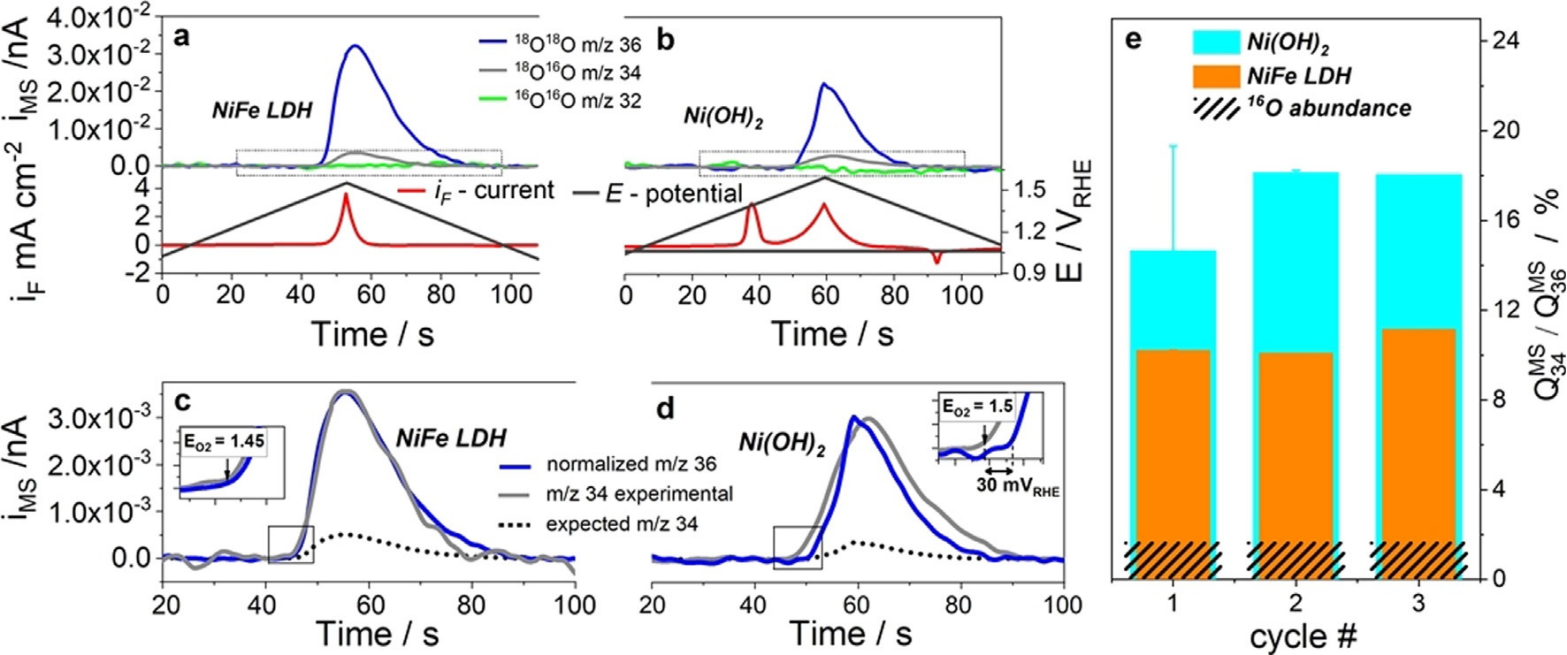
a–d) MSCV and faradic CV curves in time domain of a) ^16^O‐NiFe LDH (MSCV top, CV bottom) and b) Ni(^16^OH)_2_ (MSCV top, CV bottom) recorded in ^18^O ‐enriched 0.1 M KOH electrolyte prepared using H_2_^18^O (99.3 % ^18^O). a) and b) show the mass ion currents, i_MS_, of *m*/*z*=36 ^18^O_2_ (blue line), *m*/*z*=34 ^18^O^16^O (grey line) and *m*/*z*=32 ^16^O_2_ (green line) related to the faradaic current i_F_ (red line) and the applied potential E (black line). The experimental *m*/*z=*34 MSCVs, inside the dashed square boxes in (a) and (b), are shown enlarged in panels c) and d). Beside the experimental *m*/*z=*34 ^18^O^16^O (grey line) traces, panels (c) and (d) show the experimental mass current of *m*/*z=*36 normalized to the maximum *m*/*z=*34 mass current (dotted blue line), as well as the theoretically expected *m*/*z*=34 ^18^O^16^O ion current based on the ^16^O atomic abundance of 0.78 % in the 0.1 M K^16^OH/H_2_^18^O electrolyte (dotted black line). e) Comparison of the ratios of mass spectrometric charges of evolved ^16^O^18^O (*m*/*z*=34) and ^18^O^18^O (*m*/*z*=36) over the first three potential cycles for the Ni(OH)_2_ (cyan) and NiFe LDH (orange) (see details in Figure S5 and S6 and corresponding ^16^O abundance in Figure S7). The dashed areas corresponds to the expected ^16^O^18^O/^18^O^18^O ratio, 1.58 %, based on the ^16^O atomic abundance of 0.78 %. Error bars represent standard deviation from the average of two measurements of the same catalysts on two different electrodes.

To obtain deeper insight into the character of the OER reaction mechanism on the two Ni‐based OER catalysts, we performed DEMS experiments, during which the non‐labelled ^16^O‐NiFe LDH and Ni(^16^OH)_2_ catalysts were catalytically operated in ^18^O isotope‐labelled electrolyte, prepared from solid K^16^OH dissolved in H_2_^18^O (99.3 % abundance ^18^O) to obtain a 0.1 M solution. These experiments were conducted in the hanging droplet DEMS flow cell. The effective number of dissociated ^16^OH^−^ ions deriving from the 0.1 M K^16^OH contributed with an additional 0.18 % (ca. 500× excess) abundance to the isotope mix (see Table S1). The electrode potential was swept three times from +0.8 V_RHE_ to +1.6 V_RHE_ (inside the catalytic OER regime) and back at a scan rate of 10 mV s^−1^ and corresponding MSCVs were recorded (Figure 3).

Figures 3 a and b show the first MSCV and CV scans of the two catalysts. The prevalent molecular oxygen isotope was ^18^O_2_ at *m*/*z*=36, originating from a ^18^O^18^O coupling of two electrolyte‐derived intermediates. No *m*/*z*=32 mass signal of ^16^O^16^O was detectable. The mass signal *m*/*z*=34 (^18^O^16^O) is the most relevant for our discussions. It formed in the coupling of two distinct oxygen isotopes. The ^16^O isotope may originate from residual solvent H_2_^16^O or K^16^OH with a combined abundance of 0.78 % (balance is ^17^O); or else, and that is the key point of our discussion, from surface lattice or bulk lattice oxygen ligands present in the catalyst at the beginning of the experiment. A comparison of the theoretically expected *m*/*z*=34 (^18^O^16^O) ion current profiles derived from supporting equation S4 (Figures 3 c and d dotted black line) with the experimentally observed ^18^O^16^O profile (Figures 3 c and d, grey line) revealed a significant ^18^O^16^O ion current excess for both Ni based catalysts. For better comparison of the two experimental *m*/*z*=34 and *m*/*z*=36 MSCVs, the *m*/*z*=36 trace was normalized to the maximum intensity of *m*/*z*=34 and is shown as blue dotted line in Figures 3 c, and d. Figure 3 e displays the ratio of the experimental mass ion charges of ^16^O^18^O and ^18^O^18^O, that is, their integrated mass currents, $i_{MS}$ , for the NiFe LDH catalyst (orange bars) and the Ni(^16^OH)_2_ catalyst (cyan bars) over each cycle. The significant excess in the mixed isotope ^18^O^16^O oxygen evidences the transient participation of lattice oxygen atoms at the surface or/and the bulk of the catalyst in the oxygen evolution reaction mechanism. While the ratios in Figure 3 e is expected to decline by the gradual replacement of ^16^O by ^18^O on the catalyst surface, the low amount of lattice oxygen participating to the LOER probably requires more cycles to show the expected declining behavior. Kinetically further relevant is the cathodic shift of the onset potential of the cross‐coupled ^18^O^16^O isotope compared to ^18^O^18^O (Figure 3 c and 3 d inset). The coupling of lattice oxygen atoms with solvent oxygen atoms appears kinetically preferred, which may have to do with the ready initial availability of ^16^O ligands on the catalyst surface, while the ^18^O^18^O product requires the adsorption of two solvent molecules. The formation of *m*/*z*=32 ^16^O^16^O remained at noise level at all times, showing that the direct coupling of surface or bulk lattice oxygen atoms is unlikely.

We conclude the existence of a kinetically favored lattice oxygen evolution reaction (LOER) process on the two Ni‐based OER electrocatalysts. Note we use the term LOER regardless whether the oxygenated ligand (OH, O) from the catalyst lattice belonged to the surface or to the bulk.

To get further insight in the gradual exchange of oxygen atoms between the catalyst surface and electrolyte, we kept tracing the oxygen isotope ratios in non‐labelled electrolyte after an electrolyte exchange. Non‐labelled electrolyte was continuously flown over the catalyst surface to ensure a complete exchange of the electrolyte.

**Figure 4:**
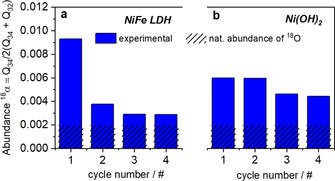
The evolution in the atomic fraction of ^18^O of the total DEMS charge of evolved oxygen measured in non‐enriched H_2_^16^O‐based electrolyte for a) NiFe LDH and b) Ni(OH)_2_. The catalysts were previously cycled into the OER range in ^18^O isotope‐enriched electrolyte. A rinsing step with non‐enriched H_2_^16^O was conducted before the experiments shown here. Shown are data obtained from MSCVs over the first four cycles (see also Figure S8 and S9). The bars represent the experimental ^18^O fraction (full blue bars) and the expected ^18^O fraction based on the natural isotope abundance (hashed bars).

Figure 4 shows the evolution of the experimental (blue bars) vs. the natural (hashed bars) atomic abundance of ^18^O during four potential cycles for both Ni‐based catalysts. The data revealed a nearly 3‐fold and more than 4‐fold higher ^18^O abundance on the first cycle for Ni(OH)_2_ and NiFe LDH, respectively, which now reflects the opposite oxygen isotope exchange between H_2_^16^O electrolyte and catalyst, following the measurements in H_2_^18^O‐based electrolyte. The absolute ^18^O isotope excess remained lower than that of ^16^O before, which indicates that only a fraction of the catalyst surface is actually contributing to the oxygen exchange processes. The faster depletion in ^18^O of NiFe LDH vs. ^18^O of Ni(OH)_2_ is fully consistent with its higher OER catalytic activity (Figure 2).

From the isotope labelling experiments, we derived the percentage of catalyst oxygen atoms that participated in the LOER mechanism. Over one potential cycle, this ratio ranged from 2.9 % for NiFe LDH to 3.6 % for Ni(OH)_2_ (see Supporting Note 3). These numbers suggest a minute contribution of the lattice oxygens of the catalysts, conceivably due to a limited accessibility of metal/oxygen moieties at the surface of the catalyst. For comparison, the ratio of electrochemically reactive Nickel atoms was evaluated from the pre‐catalytic anodic voltammetric charge under the assumption of a 1 electron transfer per Nickel center (see Supporting Note 3). For the NiFe LDH catalyst, the estimate of the electrochemically redox active Ni amounted to 1.7 % of the total Nickel atoms evidencing limited accessibility. In the case of Ni(OH)_2_, the ratio exceeded unity under the 1 electron/Ni atom assumption, evidencing redox transition from Ni^2+^ to Ni^3+^ and Ni^4+^. This analysis suggests a quite distinct electrochemical Ni accessibility between the two electrocatalysts. A role of the distinctly difference morphologies in terms of nanoplatelet size combined with a role of Fe appears plausible. This analysis is consistent with the existence of a LOER process occurring only at the surface of the nanoplatelets (and at a limited number of internal sites accessible through cracks, defects and edges of their polycrystalline domain structure^[1b]^), in agreement with the absence of bulk lattice involvement in the OER mechanism demonstrated by Roy et al.^[14]^ A bulk LOER process, involving sites deep inside the LDH interlayer regions in the center of the crystalline domains, would result in a much larger lattice oxygen contribution than that observed here.

To compare the contribution of the LOER mechanism of the Ni based catalysts with another benchmark catalyst, we performed similar isotope‐labelling DEMS OER experiments on an Iridium oxide catalyst in HCl electrolyte.^[18]^

**Figure 5:**
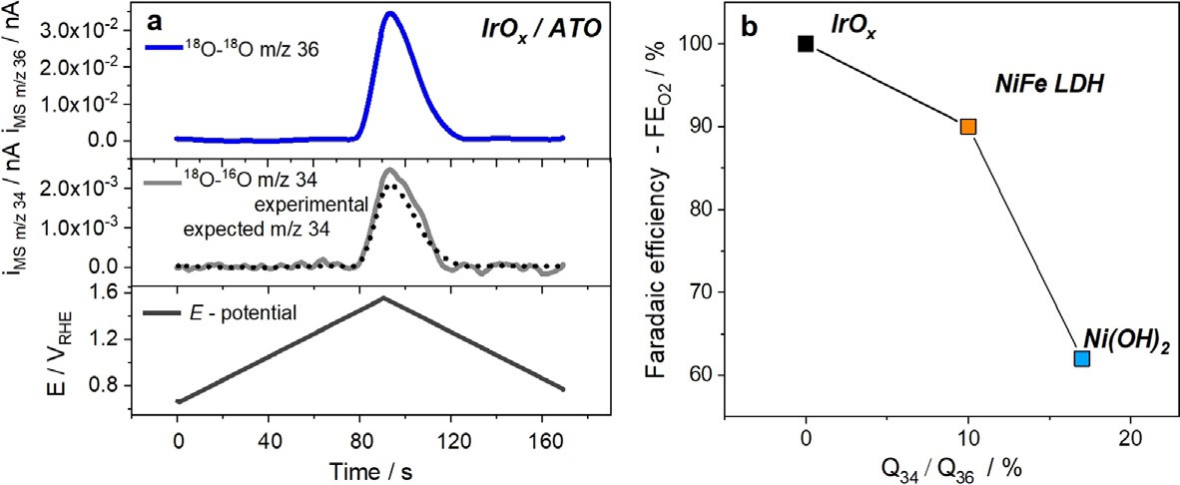
a) Mass spectrometric cyclic voltammograms (MSCVs) of the IrO_*x*_ catalyst during OER in 0.5 M HCl in ^18^O isotope labelled electrolyte (97 % abundance). Plotted are the experimental mass currents i_MS_ of *m*/*z*=36 ^18^O_2_ (blue line, top), experimental i_MS_ of *m*/*z*=34 ^18^O^16^O (grey line, center), the expected i_MS_ of *m*/*z*=34 ^18^O^16^O (dotted black line, center) based on the ^18^O abundance in the electrolyte, and the applied electrode potential sweep (black line, bottom). b) Trend in mean faradaic efficiency of O_2_ (FE_O2_), evaluated over the entire potential cycle shown in Figure 2 plotted versus ^18^O^16^O/^18^O_2_ percentage ratio of IrO_*x*_, NiFe LDH and Ni(OH)_2_. FE_O2_ data of IrO_*x*_ catalyst taken from referencer [18].

Figure 5 shows the data analysis of the Ir oxide DEMS experiments. In comparison to the theoretical *m*/*z*=34 mass current that is expected based on the ^18^O isotope enrichment of the electrolyte (dotted black line in Figure 5 a, center), the experimental *m*/*z*=34 mass current (grey line in Figure 5 a, center) was essentially a match. This evidenced a negligible contribution of lattice oxygen atoms and thus, unlike the Ni‐based catalysts, suggested no significant contribution of a LOER mechanism for the Ir catalyst.

While studies of LOER mechanisms on Ir catalysts are sparse, there are a number of LOER studies for hydrous and crystalline Ru oxides.^[20]^ A presence of LOER was reported on porous RuO_*x*_ ^[20c]^ and nanocrystalline RuO_*x*_,^[20b]^ but not on crystalline rutile RuO_2_ nanoparticles of ≈50 nm, and neither on well‐defined (100), (110), (101), and (111)‐oriented rutile RuO_2_ surfaces.^[21]^ For Ru catalysts, structural arguments were put forward, that is, LOER mechanisms are likely to occur on amorphous or hydrous phases with their large number of lattice defects, their undercoordinated sites and their high degree of redox‐active surface hydroxylation giving rise to large pseudo capacitance.^[21]^ Supporting this hypothesis further, a more pronounced presence of LOER was observed for Ru_0.9_Ni_0.1_O_2−*δ*_ than for RuO_2_:^[20b]^ Ni leaching is known to result in a lattice‐defective, redox‐active hydroxylated surface with an elevated ratio of undercoordinated sites. In view of the Ru results, the crystalline nature of our IrOx catalyst may explain the absence of a LOER mechanism.

Similar structure‐mechanism relations can be invoked to account for the presence of a LOER mechanism on NiFe LDH and Ni(OH)_2_. Even though the prepared NiFe LDH and Ni(OH)_2_ catalysts displayed bulk crystallinity (Figure S10), their catalytic active γ‐NiFe LDH and γ‐NiOOH structures feature water intercalation and hydroxylated surfaces,^[1b]^ the nanoplate morphology of which favored undercoordinated edge sites. Similar to our results, Shao‐Horn and co‐workers demonstrated LOER mechanisms in highly covalent perovskites that show pH‐dependent activity (La_0.5_Sr_0.5_CoO_3−*δ*_, Pr_0.5_Ba_0.5_CoO_3−*δ*_ and SrCoO_3−*δ*_), while less covalent and pH‐independent LaCoO_3_ lacked LOER.^[20a]^ Those perovskites contain alkaline earth metals (Ba and Sr), which easily dissolve in the electrolyte and result in the formation of surface oxyhydroxides of amorphous and, possibly, hydrous nature, with high number of undercoordinated sites, as it has been shown for BaSrCoFe perovskite.^[22]^ Finally, LOER was also observed for spinel Co_3_O_4_,^[23]^ the surface of which reconstructed under OER in amorphous CoOOH.^[24]^ Along these lines, Doyle et al.^[25]^ suggested that hydrous transition metal oxides show pH‐dependent activity. Indeed, NiFe LDH follows a (super‐Nernstian) pH‐dependence activity,[^17^, ^26^] and so does γ‐NiOOH^[27]^ and both are characterized by a LOER.

Following the most recent structural hypothesis as to the active surface site and ligand on γ‐NiFe LDH and γ‐NiOOH,^[1b]^ the successful observation of a LOER mechanism requires the presence of catalytic active surface lattice μ_2_‐OH ligands that perform a O‐O coupling with incoming electrolyte molecules. An initial, very rapid exchange of lattice OH with OH^−^ or H_2_O from the electrolyte, however, at the outset of voltammetric scans and outside the OER potential range may prevent the experimental observation of the LOER. To ensure the analytical detection of an existing LOER, the following conditions need to apply: i) the catalyst has to continuously expose pristine surface facets with not‐yet exchanged oxygen ligands due to a morphological decomposition, ii) the time of the voltammetric pretreatment should be kept at a minimum, and iii) the time resolution of the DEMS analysis has to be sufficiently high. Otherwise, failure to detect a LOER remains inconclusive.

Based on these arguments, we tend to attribute the lack of a LOER mechanism on the surface of electrochemically activated NiFe alloy nanoparticles^[14]^ to their stable bulk structure combined with rapid ligand exchange prior to DEMS detection (see Supporting Note 4), even though differences in pretreatment protocols may have a role, as well. Indeed, differences in the XAS‐determined local structure between NiFe LDH and an electrochemically Fe‐activated Ni (hydr)oxide have been recently reported by Hu and co‐workers,^[28]^ suggesting that local structural differences might exist which will have implications in the OER mechanism.

Apart from quantitative estimates of the contributions of LOER mechanisms, derived from the ratios of Q^MS^_34_/ Q^MS^_36_ in Figure 3 e, our DEMS analysis revealed another previously overlooked kinetic‐mechanistic correlation, as shown in Figure 5 b. The contribution of the LOER mechanisms of the catalysts scaled very closely with their faradaic efficiency of O_2_, FE_O2_. The catalyst with larger LOER contribution suffered from lower efficiency, that is, more holes injected in the catalyst were stored as oxidative pseudocapacitive charge in redox‐active metal centers, rather than being used to generate oxygen. Excess pseudocapacitive anodic charge, however, is known to promote undesired catalyst corrosion pathways.^[29]^ In conclusion, from a charge efficiency point of view, significant LOER contributions appear undesirable as they appear to be linked to low FE_O2_.

### DEMS Based Deconvolution of Pseudocapacitive Charge and the Effective Chemical State of Ni under OER

To learn more from the DEMS data about the chemical state of the Ni catalyst during OER, we conducted a more detailed charge analysis of the CV,$\;i_{F}$ , and the faradaic MSCV, $i_{F,\;O2}^{DEMS}$ , of β‐Ni(OH)_2_/γ‐NiO(OH). From the faradaic current density $i_{F}$ the total anodic oxidative charge $Q_{F}^{tot}$ injected into the catalyst can be estimated. $Q_{F}^{tot}$ can be deconvoluted into three different components: 1) the faradic charge $Q_{F,OER}^{DEMS}$ , (grey area) associated with O_2_ evolution from solvent molecules, 2) the faradic charge $Q_{F,LOER}^{DEMS}$ (cyan area) associated with evolution of mixed isotope O_2_ due to LOER, 3) the Ni oxidation charge, $Q_{F,Ni}$ , consumed for redox state changes of the Ni centers.

**Figure 6:**
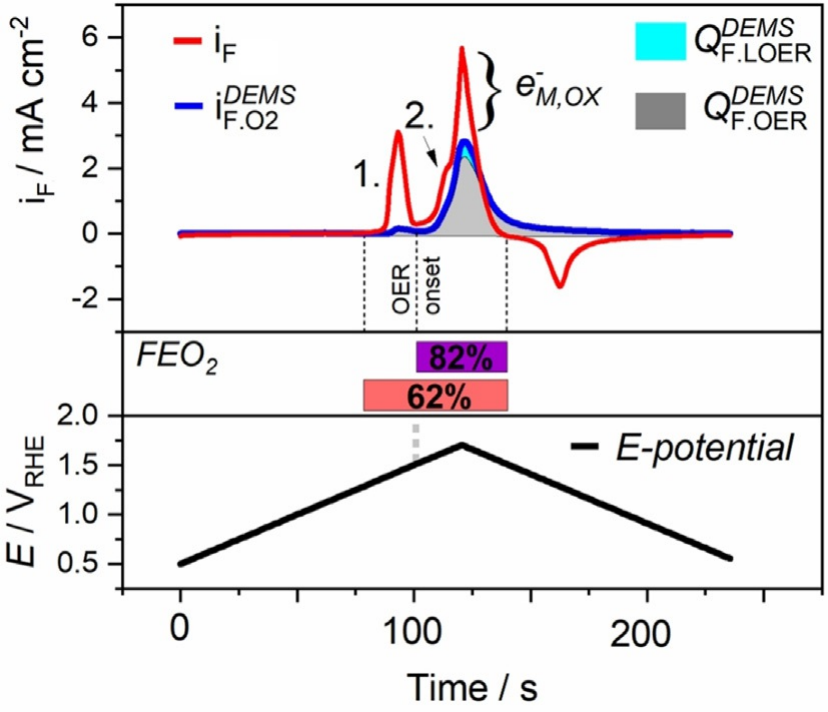
Deconvolution of the total faradaic charge under $i_{F}$ (red line) into faradaic charge and pseudocapacitive oxidative metal charge: The charge under the faradic mass spectrometric cyclic voltammogram (MSCV, blue line) of Ni(OH)_2_, $Q_{F,O2}^{DEMS}$ , splits into $Q_{F,OER}^{DEMS}$ (grey area, 86 % of $Q_{F,O2}^{DEMS}$ ) and the LOER‐derived $Q_{F,LOER}^{DEMS}$ (cyan color, 14 % of $Q_{F,O2}^{DEMS}$ . The percentage of $Q_{F,LOER}^{DEMS}$ in respect to the total $Q_{F,O2}^{DEMS}\;$ might represent a lower limit due to the exchange of surface lattice hydroxides with electrolyte). The faradaic O_2_ efficiency, FE_O2_, is dependent on the potential window considered in the integration of $i_{F}$ : FE_O2_=82 % for purple potential range, that is, without the Ni redox wave 1; FE_O2_=62 % for pink potential range including the Ni redox waves. Only anodic faradaic currents were included in the analysis to exclusively account for anodic processes (molecular O_2_).

Figure 6 shows the deconvolution of the faradaic current $i_{F}$ and the faradic mass spectrometric current $i_{F,\;O2}^{DEMS}$ for the first potential cycle (cf. Figure 3 e) and their respective charges $Q_{F}^{tot}\;$ and $Q_{F,O2}^{DEMS}$ . $Q_{F,O2}^{DEMS}$ splits into the charge $Q_{F,LOER}^{DEMS}\;$ associated with the lattice oxygen mechanism (cyan area), and into the charge $Q_{F,OER}^{DEMS}$ associated with oxygen formed from the electrolyte (grey area). The following relations for the mean faradic efficiency hold:

(1) $${{FE}_{O2}=Q}_{F,O2}^{DEMS}/Q_{F}^{tot}=\;Q_{F,O2}^{DEMS}\;/\;\left(Q_{F,O2}^{DEMS}+Q_{F,Ni}\right)\;$$

(2) $$Q_{F,\;O2}^{DEMS}=Q_{F,OER}^{DEMS}+\;Q_{F,LOER}^{DEMS}=\left({(1-x}_{LOER}\right)Q_{F,\;O2}^{DEMS}+x_{LOER}Q_{F,\;O2}^{DEMS})$$

Where $x_{LOER}$ is the percentage ratio of the lattice oxygen with respect to all oxygen. From the number ratio *λ*=Q_34_/Q_36_ (cf. Figure 3 e) we estimate $x_{LOER}$ to be 14 % (Supporting Note 3). Mean FE_O2_ values were calculated to 62 % (pink potential window in Figure 6). In other words, 38 % of $Q_{F}^{tot}\;$ is oxidative charge $Q_{F,\;Ni}$ that was injected into Ni atoms and served to increase the Ni redox state. If the Ni^+2/+3^ redox charge of peak “1” was excluded from $Q_{F}^{tot}$ by narrowing the integrated potential window, a FE_O2_ of 82 % ensued (purple potential window in Figure 6). However, this still left 18 % of $Q_{F}^{tot}$ unaccounted for, which was evidently used for the further oxidation of Ni^3+^ under peak “2”. We split the total metal charge $Q_{F,Ni}$ into the Ni^2+/3+^ transition (charge under peak 1) and the subsequent Ni^3+/4+^ transition (convoluted with the OER charge under peak 2 of $i_{F}$ ) according:

(3) $$Q_{F,Ni}=Q_{F,Ni2+/3+}+\;Q_{F,Ni3+/4+}$$

From data in Figure 6 and the relations in Supporting Note 2 we obtain

(4) $$Q_{F,Ni3+/4+}=0.6\;Q_{F,Ni2+/3+}$$

Our charge balance analysis implies the formation of Ni^4+^; more importantly, it suggests that more than half and almost 2/3 of all Ni centers of the Ni(OH)_2_ catalyst have reached the Ni^4+^ state inside the OER range. This is excellent agreement with independent measurements of the mean Ni oxidation state of +3.6 for γ‐NiOOH by previous XAS studies[^6c^, ^25^, ^30^] and fully consistent with the classical Bode redox model of Ni oxyhydroxides.^[30]^

For the NiFe LDH catalyst, the faradaic contribution of the evolved O_2_ was distinctly different (Figure S11). The DEMS‐based evaluation of $Q_{F,Ni2+/3+}$ was no longer possible, since the Ni^2+/3+^ redox process had merged with the OER voltammetric profile. Indeed, the oxidation states of Ni and Fe during catalytic OER are still being debated. Earlier XAS measurements on unsupported NiFe LDH suggested a large portion of Ni to remain in a +II state during OER, while Ni^4+^ remained below 4 %.^[31]^ By contrast, higher pH, supported catalysts or very thin catalyst films showed increased ratios of Ni^4+^. In our present study of unsupported NiFe LDH, the catalyst exhibited a large mean FE_O2_=90 %, which implied little metal redox charge, which is consistent with the low Ni^4+^ ratios reported by Görlin et al. for unsupported NiFe‐ based catalyst in 0.1 M KOH.[^11a^, ^26^, ^31^]

In summary, the DEMS‐based faradaic oxygen efficiency and charge analysis is able to deconvolute faradaic molecular oxygen charge from pseudocapacitive redox metal charge. It can be used to extract independent estimates of the chemical state of the catalyst under catalytic reaction conditions. The metal centers of the NiFe LDH catalyst appear to be in a less oxidized state compared to the Ni centers of the Fe‐free Ni(OH)_2_; however, NiFe LDH outperforms the Fe‐free catalyst in catalytic reactivity (Figure 2), which speaks to the high intrinsic activity of the NiFe LDH active sites.

## Conclusion

The present work has revealed new mechanistic aspects of the oxygen evolution process on the surface of Ni‐based OER electrocatalysts in alkaline environments.

To achieve these insights, we have first presented a versatile new differential electrochemical mass spectrometry (DEMS) liquid/vacuum cell interface, referred to as “hanging droplet cell”. The cell design addresses the need of minimum electrolyte flows where expensive isotope‐labelled reagents or solvents are involved. The usefulness of the new DEMS cell was demonstrated in the study of the OER mechanism of a Ni(OH)_2_ and a NiFe LDH catalyst in ^18^O‐labelled electrolyte.

Characteristic ^16^O^18^O isotope DEMS data suggested that the mechanism of catalytic O−O bond formation involves, to a small portion, lattice oxygen atoms at the catalyst surface. This observation was more evident in Ni(OH)_2_ than in NiFe LDH, even though valid for both catalysts. During this so‐called lattice OER (LOER) mechanism, oxygen atoms from the catalyst lattice are continuously consumed. In the present case of a LOER Mars‐Van‐Krevelen mechanism, the lattice oxygen atoms are continuously substituted by oxygen atoms from the electrolyte. In parallel to the LOER mechanism, oxygen evolves from H_2_^18^O resulting in ^18^O^18^O. A relation between LOER, faradic efficiency, the amorphous/hydrous catalyst structure, and its pH‐dependent activity is hypothesized and discussed. The case of a Mars‐Van‐Krevelen LOER mechanism has important implications for future designs or models of OER electrocatalysts that now have to consider the role and the binding of lattice atoms ligands, as well. This study highlights the importance of understanding the surface atomic structure of oxides to tune their catalytic activity.

## Conflict of interest

The authors declare no conflict of interest.

## Supporting information

As a service to our authors and readers, this journal provides supporting information supplied by the authors. Such materials are peer reviewed and may be re‐organized for online delivery, but are not copy‐edited or typeset. Technical support issues arising from supporting information (other than missing files) should be addressed to the authors.

Supplementary
