# Supplementary material for: Evidence of Mars‐Van‐Krevelen Mechanism in the Electrochemical Oxygen Evolution on Ni‐Based Catalysts
Source: Angew Chem Int Ed Engl. 2021 May 26;60(27):14981–8. doi: 10.1002/anie.202101698 (PMC8251801; doi:10.1002/anie.202101698)
Supplement: Supplementary file 1 — Supplementary [file ANIE-60-14981-s001.pdf]

Angewandte Chemie
Eine Zeitschrift der Gesellschaft Deutscher Chemiker

Supporting Information

Evidence of Mars-Van-Krevelen Mechanism in the Electrochemical Oxygen Evolution on Ni-Based Catalysts

*Jorge Ferreira de Araújo, Fabio Dionigi, Thomas Merzdorf, Hyung-Suk Oh, and Peter Strasser**

anie_202101698_sm_miscellaneous_information.pdf

1

2

1. Experimental Methods

1.1 Catalyst Synthesis

A Ni-Fe layered double hydroxide (LDH) catalyst (Ni:Fe=3.55:1) was prepared using a solvothermal synthesis reported elsewhere. [1] A reference $Ni(OH)_2$ catalyst was synthesized in a two-step process: In the first step, hydrous $Ni(OH)_2$ nanoparticles were precipitated using $Ni(II)(OAc)_2$ and 1 M KOH. This suspension was treated hydrothermally, washed and then freeze-dried to obtain $Ni(OH)_2$. All chemicals were used as received. A 0.1 M KOH electrolyte (semiconductor grade, 99.99% on trace metals basis, Sigma-Aldrich) was prepared from $H_2{}^{18}O$ (^{18}O abundance 99.3%) obtained from Sercon Ltd Isotopes, UK. All other chemicals were purchased in analytical grade quality and used as received. Fe-free purified electrolytes solution was processed through an additional purification step according to the method reported by Trotochaud et al. [2]

1.2 ^{18}O enriched electrolytes

For the preparation of ^{18}O enriched 0.1 M $K^{16}OH$ solution, a glove box was used to weight an appropriate amount of $K^{16}OH$ salt followed by dissolution in $H_2{}^{18}O$. The ^{18}O

abundance was 99.3% for the analysis of NiFe layered double hydroxide (LDH) and $Ni(OH)_2$ catalysts. A 97% abundance $H_2{}^{18}O$ was used for the preparation of an ^{18}O enriched 0.5 M HCl electrolyte for the measurements and analysis of Ir oxide catalyst.

1.3 Use of small volumes of isotope-labelled solvent ($H_2{}^{18}O$)

DEMS based OER studies are often done on non-isotope-enriched catalysts in isotope-enriched solvents, such as $H_2{}^{18}O$, to analyze the isotopologues of the evolving molecular oxygen. This gives insight in the presence of either an edge-site-mediated O−O bond formation via a direct intramolecular coupling of O ligands, or the coupling of a catalyst lattice oxygen ligand with a solvent molecule (lattice oxygen evolution reaction, LOER). DEMS is an ideal tool to investigate the presence of such mechanisms in isotope enriched $H_2{}^{18}O$ based electrolytes. The main difficulty is the use of a lower volume of electrolyte during flow cell measurements, due to the high cost of $H_2{}^{18}O$. In a normal situation, DEMS analysis during OER would use between 30-40 ml of the electrolyte solution. The design of a new hanging droplet cell, as detailed below, enabled DEMS measurements with a fraction of that electrolyte volume.

1.4 Electrode preparation and test protocols

Polished glassy carbon electrodes (Ø5 mm, HTW GmbH) were partially covered by a Teflon mask such that an area enclosed by a circle of 3 mm diameter was exposed onto which the unsupported NiFe LDH and $Ni(OH)_2$ catalysts were loaded at a 10 $\mu g_{(Ni+Fe)}$ cm^{-2} loading.

For conventional, non-labelling experiments, the dual thin-layer cell was used (**Figure 1a**). The purified 0.1 M $K^{16}OH$ electrolytes (pH 13) was continuously purged with argon (5.0 N) in an external vessel, and pumped through the cell at a flow rate of 5 µl s^{-1} using hydrostatic Ar pressure above the electrolyte. A voltammetric activation protocol was carried out, which involved scans between +0.5 V_{RHE} and a current density of 6 $mAcm^{-2}$ at a scan rate of 10 mVs^{-1} until a stable CV was reached (~ 20 cycles). The catalyst electrodes were then allowed to further stabilize in the electrolyte solution at 1.1 V_{RHE}, in order to record the ionic current baseline before recording the mass spectrometric cyclic voltammetry cycles. Mass spectrometric cyclic voltammograms (MSCV) were then recorded between +0.5 V_{RHE} and the potential at which a current of 6 $mAcm^{-2}$ was reached (**Figure 2**) at a scan rate of 5 mVs^{-1} with a continuous flow of fresh electrolyte over the catalysts. The DEMS-derived OER onset

potentials were evaluated as the electrode potential at which the oxygen mass ion current reached 1% of the oxygen mass ion current maximum.

The hanging droplet flow cell (**Figure 1b**) was used in the isotope-enriched DEMS studies involving 0.1 M KOH in $H_2{}^{18}O$. The $H_2{}^{18}O$ solvent was used without further purification. The Ar saturated electrolyte was loaded in a gas-tight syringe. Using a syringe pump, the electrolyte was then pumped through the cell at a flow rate of 1 μl s^{-1}. The $H_2{}^{18}O$ isotope analysis is given in **Table S1**. The ^{18}O isotope enriched experiments in the hanging droplet flow cell were preceded by an activation protocol using the non-^{18}O enriched 0.1 M KOH electrolyte solution. The catalyst was cycled in non-isotope labelled 0.1M $K^{16}OH$ in $H_2{}^{16}O$ for the purpose of catalyst activation for a total of 10 cycles between 0.8 – 1.5 V_{RHE}. After the catalyst activation, the electrolyte solution was removed, and the catalyst-coated electrode left to dry under Argon gas flow. For the isotopes experiments with ^{18}O enriched electrolyte, the dry catalyst electrode was connected to the potentiostat and kept at open-circuit potential, while the aqueous ^{18}O -enriched 0.1M $K^{16}OH$ electrolyte was filled into the electrochemical cell for subsequent DEMS analysis. The potentiostat was set to an initial electrode potential between 0.8 V_{RHE} and 1.0 V_{RHE} and the potential was swept up to +1.6 V_{RHE} and back at a scan rate of 10 mVs^{-1} for several cycles.

1.5 Faradaic product efficiency

Formation of volatile products (*m/z* 2, 16, 18, 28, 30, 36, 32, 34, 44) were monitored during voltammetric potential scans. Evaluation of faradaic efficiencies of O_2 required a product-specific calibration constant (K^*) obtained from quasi-stationary state calibration measurements, where constant faradaic currents, i_F, were applied, while recording ion mass currents after 30 sec. The unitless calibration constant K^* relates the applied faradaic current (i_F) with the corresponding mass spectrometric current (i_{MS}) of species j in a given cell environment according to [3]:

$$K_j^* = \frac{i_{MS,j} \cdot n_j}{i_F} \qquad \text{(S1)}$$

where n_j represent the number of transferred electrons per molecule of product j. The K^* value (in this work for O_2: 8.99 10^{-6} for $Ni(OH)_2$ and 8.59 10^{-6} for NiFe LDH) incorporates the Faradic constant, F, and is used to convert experimental mass spectrometric ion currents, $i_{MS,j}$, or charges, $Q_{MS,j}$, of new catalysts into their corresponding DEMS-derived faradaic

charges, $Q_{F,j}^{DEMS}$. The faradaic efficiency of product j averaged over the time of a voltammetric scan was calculated by integration of mass spectrometric and faradaic currents in the time domain resulting in a mass charge, $Q_{MS,j}$, and the total faradaic charge, Q_F^{tot}. Charges and the number of transferred electrons per molecule, n_j, yield the efficiency according to [4]

$$FE_j(\%) = \frac{Q_{MS,j} \cdot n_j}{Q_F^{tot} \cdot K_j^*} \cdot 100 = \frac{Q_{F,j}^{DEMS}}{Q_F^{tot}} \cdot 100. \qquad \text{(S2)}$$

Here, $Q_{F,j}^{DEMS}$ denotes the DEMS-derived faradaic charge that is converted into product j. Differential faradic efficiencies are obtained from the respective currents.

1.6 Atomic O isotope abundances

For the interconversion between atomic O isotope abundances and DEMS-derived O_2 isotopologue charges, the mass ion currents of $^{16}O_2$, $^{18}O^{16}O$, and $^{18}O_2$ molecules were recorded and analyzed. The atomic abundance of ^{16}O-atoms, $^{16}\alpha$, in ^{18}O-enriched electrolyte, neglecting the minute contribution of $^{16}O_2$ mass charges, follows from the integrated DEMS-derived charges as:

$$^{16}\alpha = \frac{Q_{MS,^{16}O^{18}O}}{2\left(Q_{MS,^{16}O^{18}O} + Q_{MS,^{18}O^{18}O}\right)} \qquad \text{(S3)}$$

Conversely, the expected ratio of O_2 mass ion charges is obtained from a given atomic abundance using:

$$\frac{Q_{MS,^{16}O^{18}O}}{Q_{MS,^{18}O^{18}O}} = \frac{2\ ^{16}\alpha}{(1-2\ ^{16}\alpha)} \qquad \text{(S4)}$$

Comparisons of expected and experimental isotope abundances were used to test the presence of catalyst lattice oxygen participation in the OER process.

1.7 The Differential Electrochemical Mass Spectrometry (DEMS) system

The DEMS setup incorporated two serial vacuum chambers: the primary high-vacuum mass spectrometer (MS) chamber (Pfeiffer PrismaPlus® QMG 220) and a second chamber that served as an intermediate vacuum region (~10^{-3} mbar) between the MS chamber and

electrochemical cell. The Pfeiffer MS chamber was connected to the intermediate chamber using a Ø 6 mm open flange (**Figure S1**). A turbomolecular pump (Pfeiffer HiPace® 80) was mounted directly in each stage and backed by a diaphragm pump and rotary vane pump. The system design allows the pressure reduction over the two stages from 10^{-3} mbar down to 10^{-6} mbar in the MS chamber.

1.8 Details on DEMS apparatus – Analysis QMS chamber (second stage chamber)

The primary purpose of the analysis chamber (second stage chamber in **Figure S1**) is to provide optimal conditions to run the MS detectors. The MS detectors are the Faraday cup and the secondary electron multiplier (SEM). The analysis chamber has an internal diameter of 60 mm and enough length to place in the quadrupole mass spectrum (QMS) instrument. The MS system is composed by a gas-tight ion source with two W-filaments and a quadrupole mass filter with a rod diameter of 6 mm and length 100 mm (Pfeiffer QMA 200). The inlet of the analysis chamber is done by a 6 mm diameter hole drilled in a DN40 flange with thickness 10 mm. The incoming gas analyte is introduced directly into the ionization area by placing the DN40 flange inlet hole in direct connection with the metal tube belonging to the ion source, with an insulating ceramic tip electrically insulating the two. The turbomolecular pump (TMP) at the analysis chamber is located in the middle of the analyzer (underneath the quadrupole rods) promoting high vacuum (10^{-6} mbar) and a molecular flow regime. High vacuum is necessary to allow ions to reach the detector without undergoing collisions with other gaseous molecules. Indeed, the collision would produce a deviation of the trajectory, and the ion would lose its charge against the walls of the instrument.

On the other hand, ion-molecule collisions could produce unwanted reactions and hence increase the complexity of the spectrum. Mass spectrometric data is collected with Quadera software. At this point, it is essential to realize that pressure in the 10^{-6} mbar range – necessary for incoming flux and low enough to run the SEM detector under well-defined conditions – requires the whole chamber to be baked to 100-150°C. Heating the chamber cannot be done while running degassed reaction products because it would promote pressure oscillation due to the high amount of water present. Therefore, the heating is done before and with a close degassing valve, which allows reestablishing reproducible conditions by the MS instrument.

1.9 Pressure gradient generation in the first stage vacuum chamber

The pressure gradient generation chamber (first stage vacuum chamber) is an important component of the DEMS system as any other component. It promotes a differential pressure and continuous high flow of the degassed reaction products in a vacuum while running mass spectrum in simultaneously (**Figure S1**). The pressure gradient generation region is composed by a gas dosing valve connected to a DN 40 CF Tee. The generated vacuum in this region is in the range of rough-medium, which is enough to maintain degassing processes and to assure continue pressure gradient over time. The magnitude of the pressure gradient depends upon the conductance of the system ($S_1 = S_2 = 67\ l\ s^{-1}$), rate of outgassing and flux from the flow cell, leak rate, the pump capacity and the distance between the pumps. The incoming flow inside the vacuum chamber contains a large percentage of water vapor. The turbo molecular pump (TMP) works in parallel with a baked system composed by two rough vacuum pumps; a rotary vane pump (RVP), first stage, and diaphragm pump, second stage.

1.10 The dual thin-layer electrolyte flow cell

The dual thin-layer electrolyte flow cell (**Figure 1a**) consists of two horizontal parallel disk-shaped compartments with connecting liquid channels. [5] The thin-layer electrolyte compartments are typically a couple of hundred micrometer-thick (~100 µm). Mimicking a wall-jet Ring-Disk electrode setup, the electrolyte enters the reaction zone flowing up against the electrode interface and exiting by flowing radially outward across the horizontal, disk-shaped region thereby passing over the reactive interface. Four thin channels originate from the edge of the top thin-layer compartment and guide the electrolyte down to the second disk shaped compartment where the interface to the vacuum is. The radial flow is essential for avoiding the formation of “dead” volume. In the top thin-layer compartment, the entrance is built by the intersection of two perpendicular channels, which connect in one point at the center inlet. One channel directs a large portion of the incoming flow of electrolyte to the reaction zone and a smaller portion to a second perpendicular channel, where a platinized platinum wire counter electrode (CE), prepared by electro-deposition of a 0.5 M $HPtCl_6$ solution on the platinum wire, was placed. A home-made reversible hydrogen electrode, RHE, made of a Pt wire that was constantly replenished with H_2 gas, served as reference electrode (RE).

1.14 The new hanging droplet flow cell design

The hanging droplet DEMS flow cell (**Figure 1b**) allows working with microliter scale electrolyte volumes (typically 20-50 µl). This capability is useful when small, expensive electrolyte volumes are concerns, such as isotope-labelled compounds. Thus, for all the isotope labelled OER experiments the droplet flow cell is used. The electrochemical measurement is maintained under convection conditions, and a constant liquid droplet volume is maintained. The ability for visual inspection of the electrode and electrolyte contact helps removal of undesired gas bubble formation by controlled flushing using a tube in a direction perpendicular to the surface of the electrode. For the flow of the electrolyte stream, a syringe pumping is employed to maintain constant the inlet flow at 1 µl s^{-1}. The concentric larger-diameter electrolyte inlet tube is placed at 2 mm from the electrode surface. The inner concentric product withdrawal is achieved using a Ø150 µm inner diameter capillary placed concentric inside the inlet tube but as close as 300 µm from the electrode surface. The setup is gastight and an absolute pressure of 2.5 bar Argon is maintained and controlled above the electrolyte by a high precision pressure gauge. The counter electrode is a Ø0.5 mm platinum wire and a leak-free Ag/AgCl is used as reference electrode with a Ø1 mm shaft (Warner Instruments), **Figure 1**. The reaction products are collected through the capillary together with electrolyte at 1 µl s^{-1} and introduced to a flow splitter that directs the flow to the disk-shaped liquid/vacuum chamber via four thin channels. In the disk-shaped liquid/vacuum chamber a PTFE membrane makes the interface between liquid and vacuum. This compartment has an inward radial electrolyte flow pattern because the four thin channels connect to the outer edges of the liquid/vacuum chamber. The stationary circulation of electrolyte is achieved by a single exit hole in the center of the liquid/vacuum chamber **(Figure S2a).**

The minimum useable volume of electrolyte of the hanging droplet flow cell is between 20-50 µl. This is enough to interface the active electrode surface area of 3 mm diameter. The droplet 3D shape allows contact with a reference and counter electrode, as well as the concentric inlet /outlet flow tube. Another important advantage of the extremely low volume of electrolyte is the fact that it ensures a high volumetric concentration of products in the analysis and allows for low faradic currents. During the electrochemical measurements, a constant volume of the hanging droplet is maintained by the stationary in/outflow of electrolyte under convection conditions. The glass cell allows visual inspection of the

electrode and electrolyte contact. This setup requires a volume of $H_2{}^{18}O$ of about 1.5 ml to run several voltammograms.

1.15 Electrolyte flow profile

The two DEMS flow cells used in this study (Hanging droplet flow cell, dual thin-layer flow cell) have a uniform flow pattern across the electrode surface. Both flow cell systems are presented in **Figure S2** with a representation of the possible flow patterns circulating over the electrode surface. The gas products are transported to the liquid/vacuum interface. The continuous electrolyte flow over the surface minimized diffusional transport limitations at the electrode surface and thereby maintained a sustained current without gas bubble formation.

1.16 The computational fluid dynamics (CFD) analysis of the dual thin-layer flow cell

One very important, distinctive characteristic of the present dual thin-layer flow cell design (**Figure S3a**) is the homogeneity of the shear stress across the electrolyte/electrode interface, which prevents the formation of detrimental gas bubbles. Gas bubbles are detrimental because the presence of the gas phase would promote variations in the faradaic current distribution over the electrode surface.

Computational fluid dynamics (CFD) was used as a helpful tool to model the shear stress gradients across the electrode interface (**Figure S3b,c**). **Figure S3b,c** shows modeling data comparing the original dual thin-layer cell design, equipped with one central electrolyte inlet tube and four symmetric edge capillary flow channels feeding into the lower thin-layer compartment, with two new superior designs (design 2, design 3). Modeling data suggest that design 3 offers the optimal shear stress gradient and distribution conditions characterized by a very uniform shear stress distribution over the electrode interface.

2. Supporting Figures

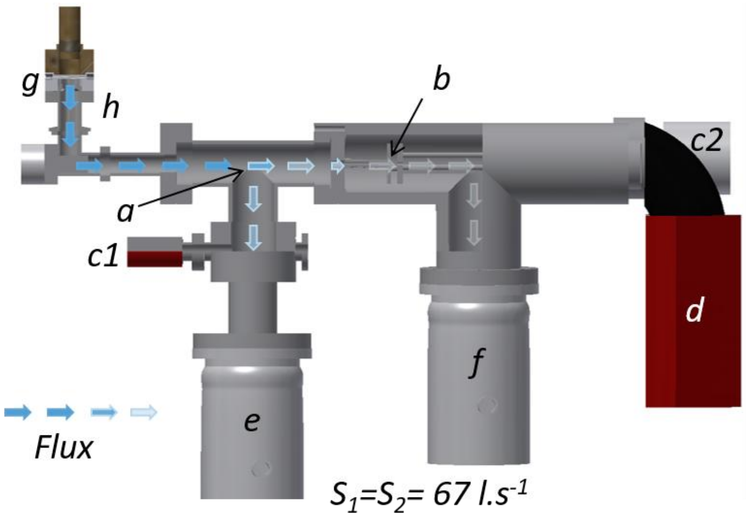

Figure S1 Cross-section view of the DEMS set up with gas product flow paths indicated by arrows. DEMS system consists of two vacuum chamber stages. **a)** First stage vacuum chamber, **b)** Second stage vacuum chamber with ionization area at the gas-tight ion source connected to the quadrupole mass spectrometer (QMS), **c1** and **c2)** pressure sensors, **d)** electronics (QME), **e** and **f)** turbo molecular pumps (TMPs) pumping at comparable S_1, S_2 volumetric pump rates, **g)** the dual thin-layer flow cell, **h)** Connecting flange to first stage vacuum chamber.

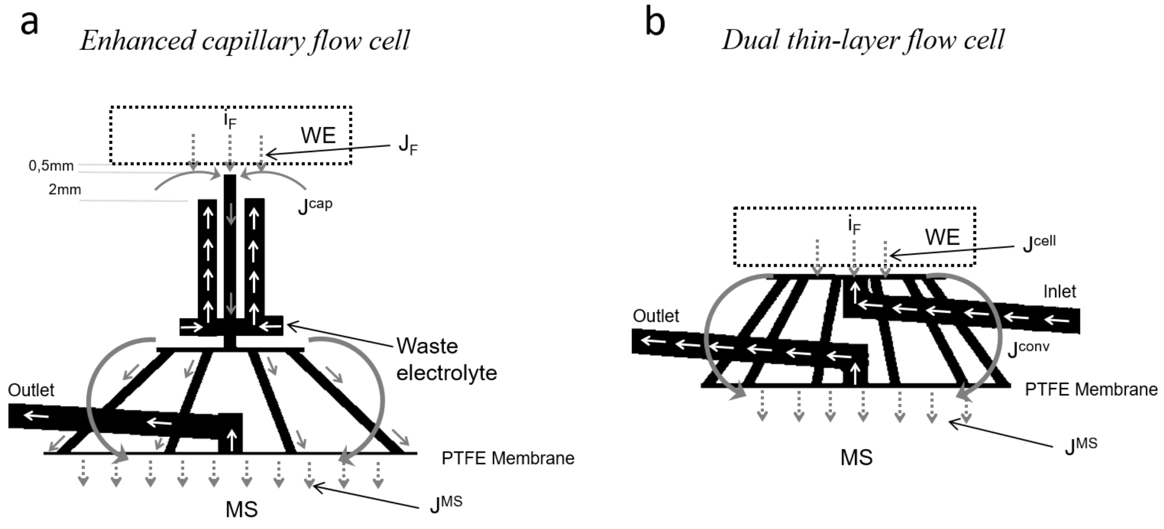

Figure S2 Schematic representation of side view of the main flow path with intense convection flow of electrolyte over the membrane and thin-layer compartments (represented in dark). The products saturated flow direction is represented by the full grey arrows (J^{conv}) and the products flow at the electrode surface and membrane interface are represented by dash grey arrows (J^{cell} and J^{MS}); **a)** enhanced capillary flow cell, **b)** dual thin-layer flow cell. WE, working electrode, MS mass spectrum and i_F faradaic current.

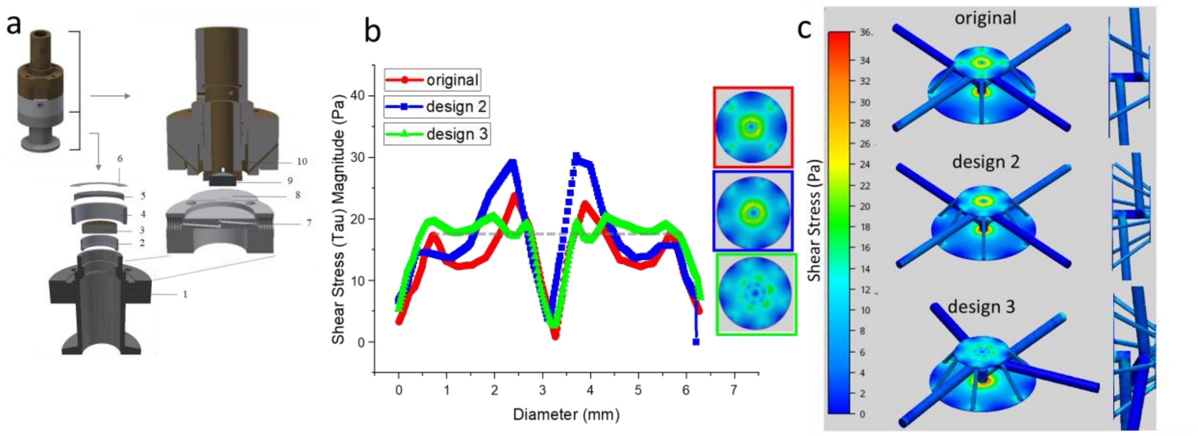

Figure S3 Study of flow cell design on applied shear stress forces at the catalyst surface. a) schematic diagram of the electrochemical dual thin-layer flow cell: **1)** – down support, **2)** support frit material, **3)** frit material, **4)** ring for PTFE membrane, **5)** PTFE membrane, **6)** down PTFE spacer, **7)** flow channels, **8)** top PTFE spacer; **9)** working electrode (WE); **10)** support for WE. b) Shear stress magnitude (in Pa) of three distinct dual thin-layer designs (original, design 2, design 3, component 7 of a)) versus the distance from one edge side channel to the opposite edge side channel (distance along the diameter in mm). Simulations of the shear stress distributions of the electrolyte flow through the top thin-layer are shown as top view in the three insets of b), insets are framed using the same colors as the corresponding curves in b), and are shown as 3D models in c). In c) the side views of the 3D models are shown on the right of the corresponding 3D models. Original design [5]: inlet Ø1 mm and 4 edge disk capillary channels Ø0.5 mm, design 2: inlet Ø1mm and 6 edge disk capillaries Ø0.4 mm and design 3: total of 7 inlets (6 x Ø0.4 mm and 1 x Ø0.7 mm) and 6 edge disk capillaries Ø0.5 mm. An internal pressure drop of 0.05 bar is assumed. The calculated shear stress distribution inside the functional part in Figure S3a "7". The warmer the color the higher the shear stress. Study done on Autodesk CFD software with parameters; Inlet pressure of 2 bar; Outlet pressure or backpressure of 1.8 bar; Outlet flow 5 µl s^{-2}.

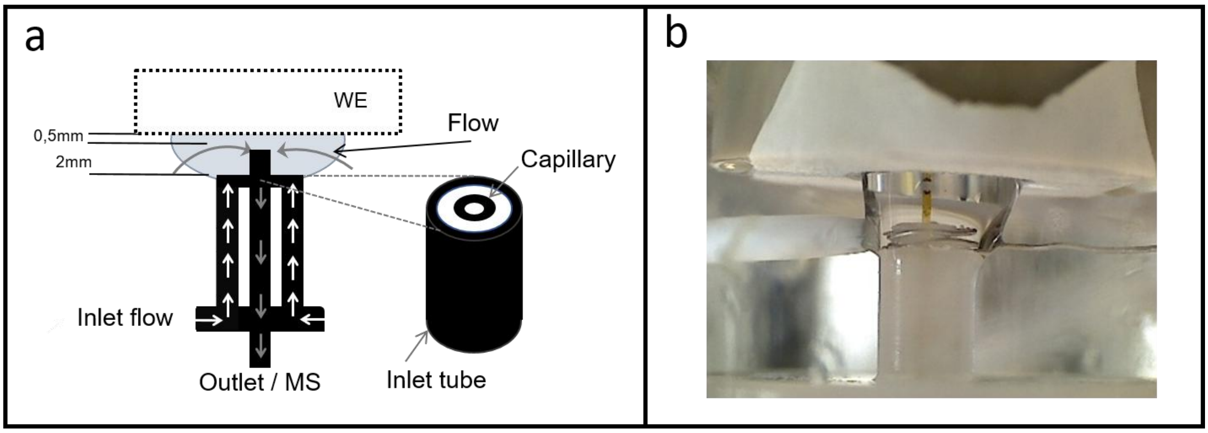

Figure S4 a) Schematic representation of side view of the main flow path in the droplet flow cell. The products saturated flow direction is represented by the full grey arrows. b) Photography of a droplet flow cell apparatus.

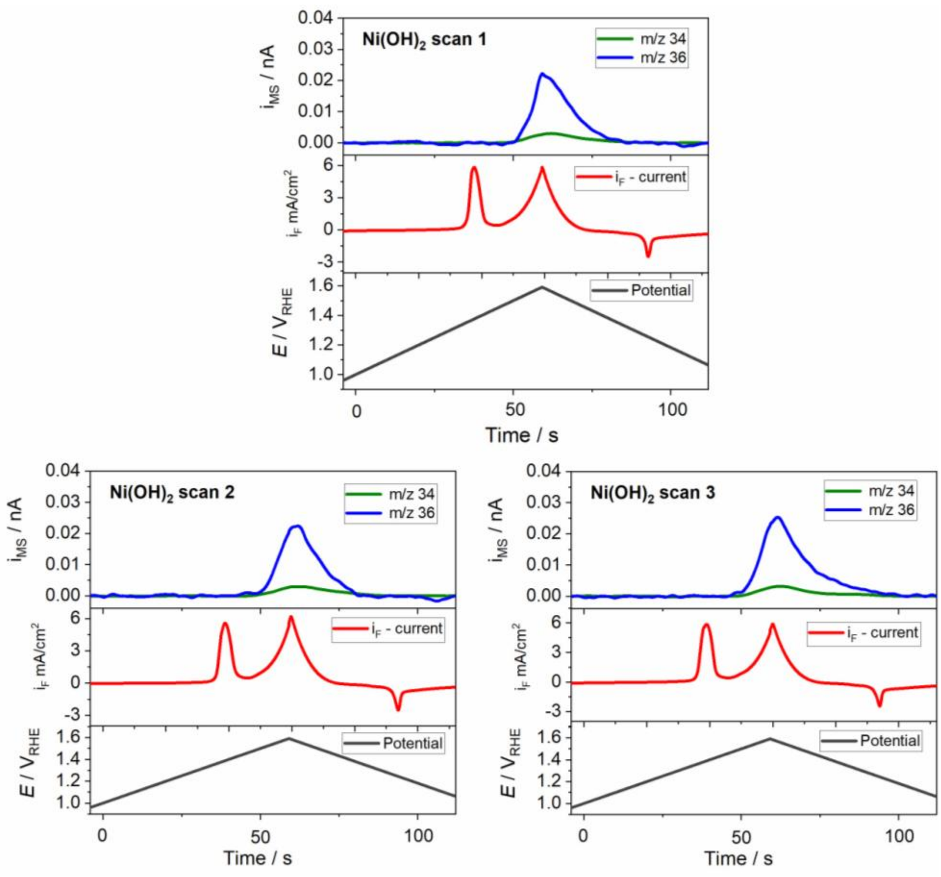

Figure S5 Mass spectrometric cyclic voltammogram curves (MSCV), i_{MS} , in time domain for $Ni(OH)_2$ catalysts in electrolyte solution of 0.1 M KOH made of 99.3% $H_2{}^{18}O$. The mass spectrum curves for m/z 36 isotope $^{18}O_2$ (blue line) and m/z 34 curves representing the mass signal of $^{16}O^{18}O$ (green line), the faradaic current i_F – mA cm^{-2} (red line) and the potential wave program (black line) are shown for the first three potential scans.

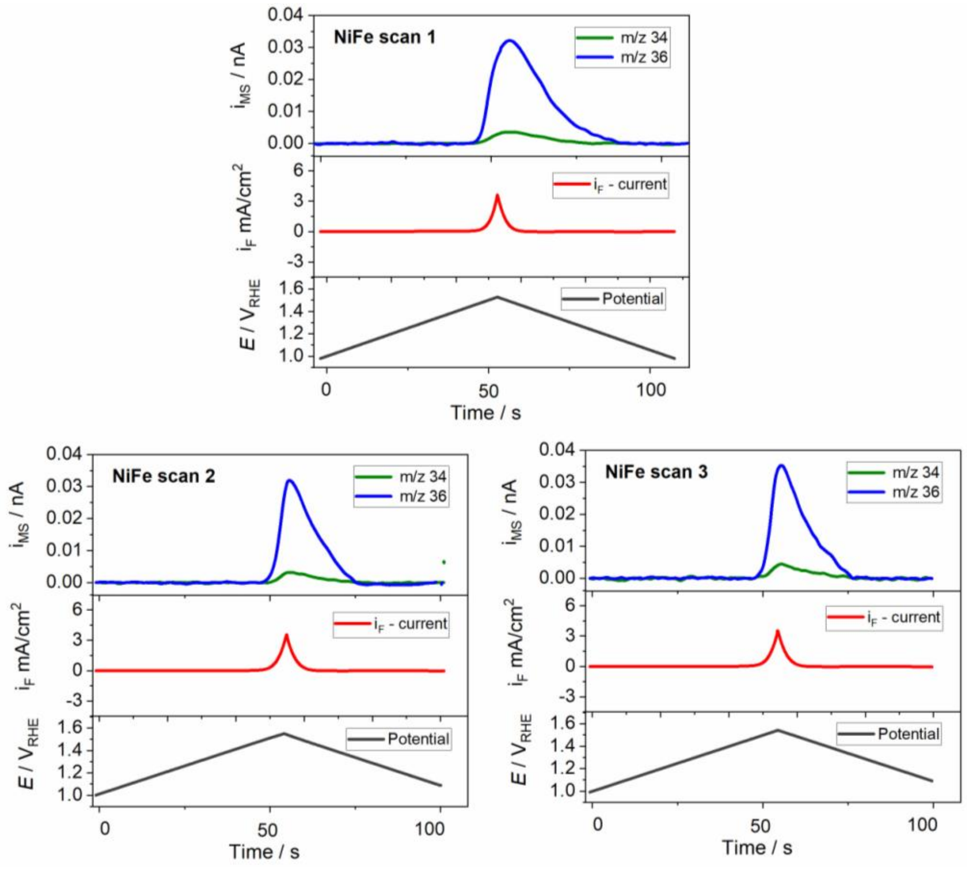

Figure S6 Mass spectrometric cyclic voltammogram curves (MSCV), i_{MS}, in time domain for NiFe LDH catalyst in electrolyte solution of 0.1 M KOH made of 99.3% $H_2{}^{18}O$. The mass spectrum curves for m/z 36 isotope $^{18}O_2$ (blue line) and m/z 34 curves representing the mass signal of $^{16}O^{18}O$ (green line), the faradaic current i_F – mA cm^{-2} (red line) and the potential wave program (black line) are shown for the first three potential scans.

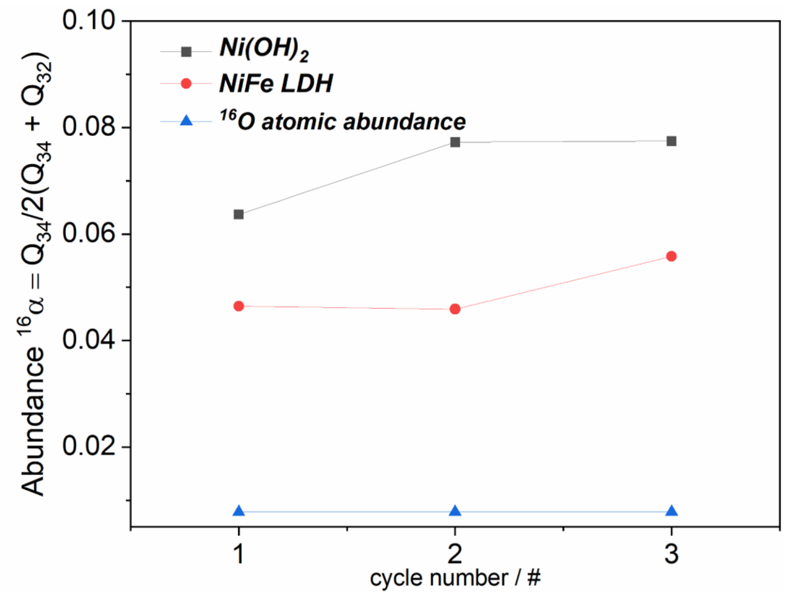

Figure S7 The evolution in the atomic fraction of ^{16}O of the total DEMS charge of evolved oxygen measured in isotope enriched $H_2{}^{18}O$-based electrolyte for NiFe LDH and $Ni(OH)_2$.

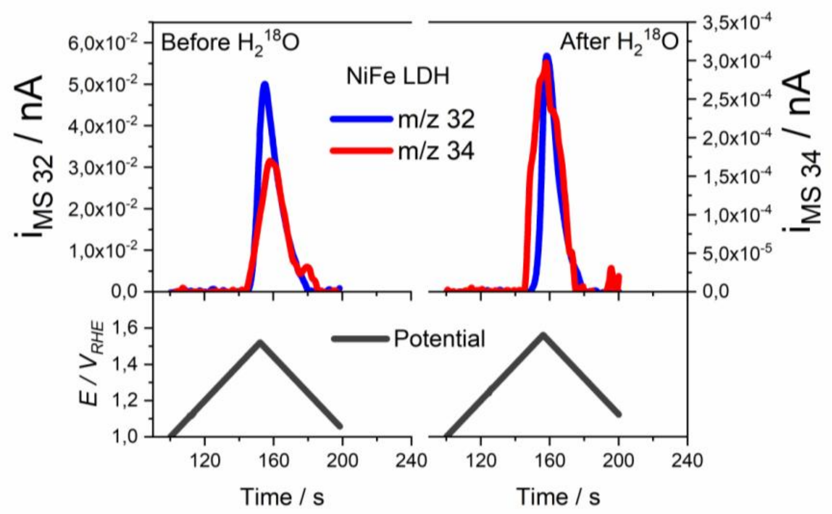

Figure S8 Mass spectrometric cyclic voltammogram curves (MSCV), i_{MS}, of m/z=32 and 34 for the NiFe LDH catalyst plotted in the time domain. Left) during the cyclic voltammogram activation in 0.1 M $K^{16}OH/H_2^{16}O$ before the contact with 0.1 M $K^{16}OH/H_2^{18}O$ electrolyte, and Right) in in 0.1 M $K^{16}OH/H_2^{16}O$ but after the experiment in 0.1 M $K^{16}OH/H_2^{18}O$ electrolyte.

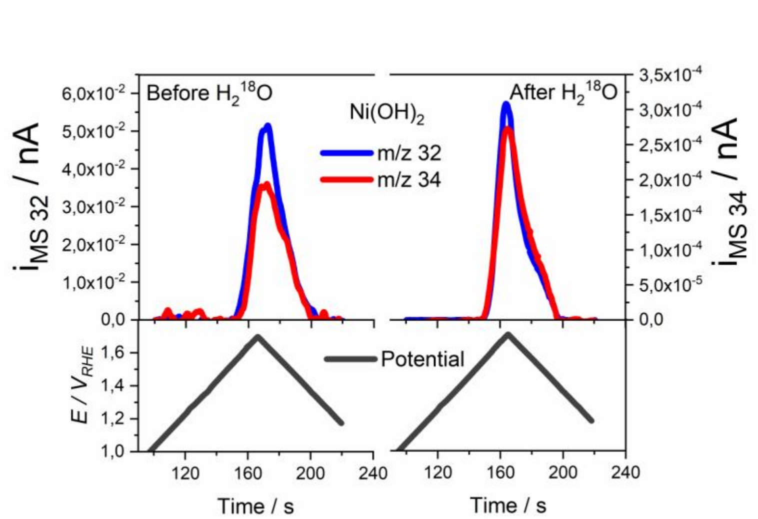

Figure S9 Mass spectrometric cyclic voltammogram curves (MSCV), i_{MS}, of m/z=32 and 34 for the $Ni(OH)_2$ catalyst plotted in the time domain. Left) during the cyclic voltammogram activation in 0.1 M $K^{16}OH/H_2^{16}O$ before the contact with 0.1 M $K^{16}OH/H_2^{18}O$ electrolyte, and Right) in in 0.1 M $K^{16}OH/H_2^{16}O$ but after the experiment in 0.1 M $K^{16}OH/H_2^{18}O$ electrolyte.

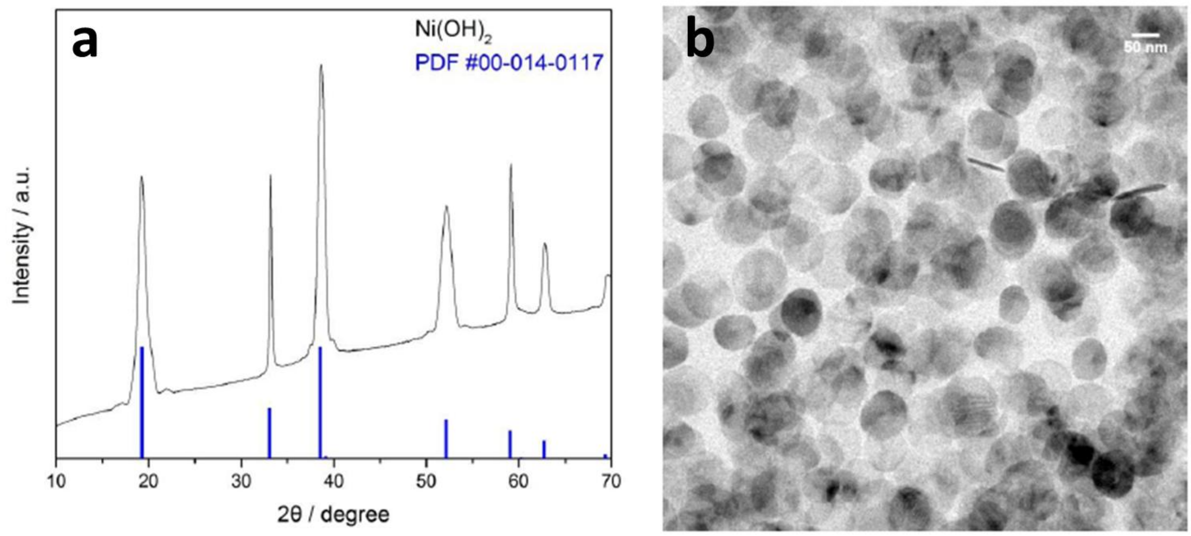

Figure S10 a) XRD pattern of $Ni(OH)_2$, with the reference profile PDF #00-014-0117 suggesting β-$Ni(OH)_2$ as the non catalytic rest phase. b) TEM micrograph of β-$Ni(OH)_2$.

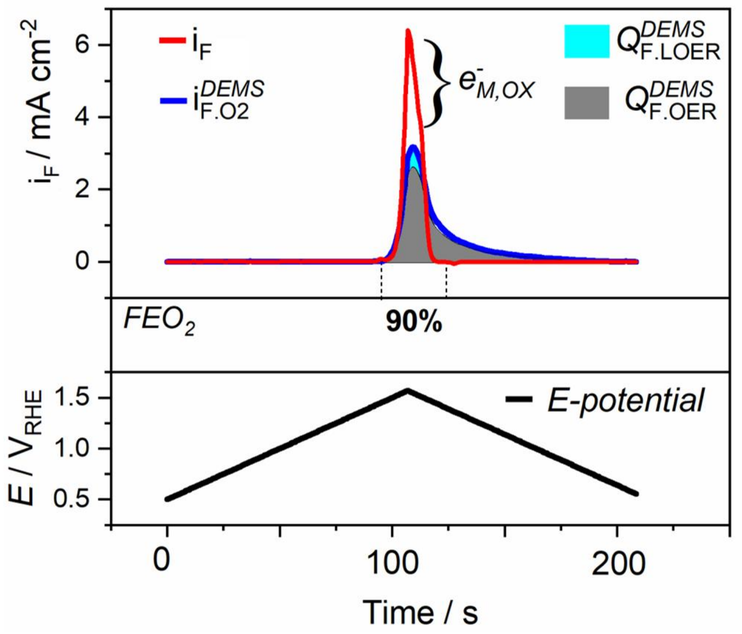

Figure S11 Representation of the two deconvoluted faradaic current derived from mass spectrum signals for NiFe LDH. The two components of $Q_{F,O2}^{DEMS}$, namely $Q_{F,OER}^{DEMS}$ (grey area) and LOER $Q_{F,LOER}^{DEMS}$ (cyan area), where ($x_{LOER} = 9\%$ of $Q_{F,O2}^{DEMS}$), all in in mA cm^{-2}. Only anodic faradaic currents were included in the analysis to exclusively account for anodic processes (molecular O_2). The total experimental faradaic current is given by i_F (red line) curve. The faradaic efficiency, FE_{O2} = 90%, is shown in a red bar below the curves and includes the contribution of metal redox processes.

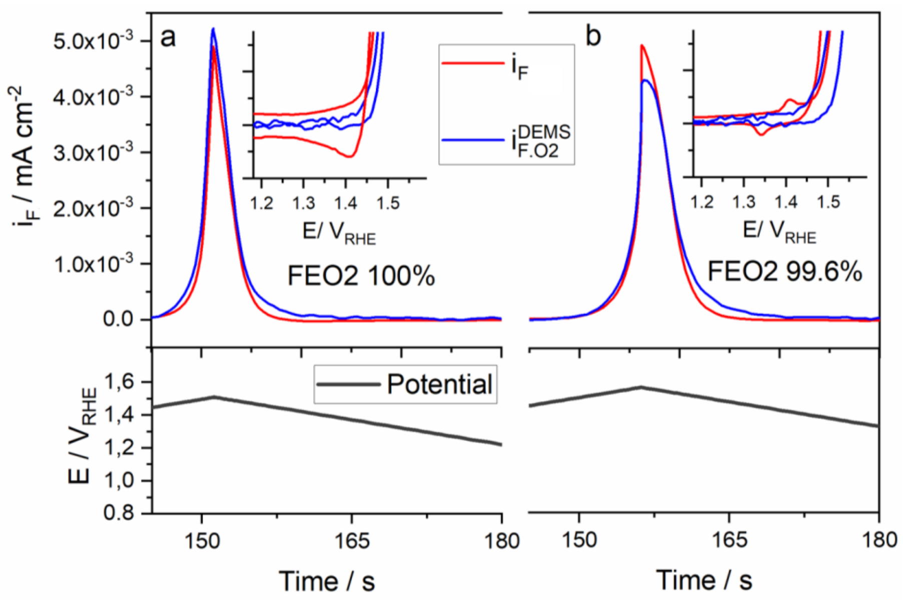

Figure S12 Non isotope-labelled DEMS analysis of the OER process on a) a polished polycrystalline Iridium metal disk and on b) a polished polycrystalline Iridium metal disk with a Ni monolayer electrodeposited on the surface [6], both in Ar- saturated 0.1 M KOH electrolyte. The faradaic mass spectrometric cyclic voltammogram (MSCV), $i_{\mathrm{F,O2}}^{DEMS}$, of m/z=32 $^{16}O_2$ (blue line) was obtained from the raw mass ion MSCV, i_{MS}, by conversion using equation S2 and is plotted along with the electrochemical faradaic current i_F (red line) and the cyclic potential E scan (black line). Both faradaic MSCV and faradaic CV are plotted in units of mA cm^{-2}.

3. Supporting Tables

Table S1. Oxygen isotope abundance in the $H_2{}^{18}O$ used in this study. Data obtained by certified analysis Taiyo Nippon Sanso.

Test	Unit	Test
Oxygen-^{18}O	Atom%	99.3
Oxygen-^{17}O	Atom%	0.1
Oxygen-^{16}O	Atom%	0.6
Oxygen-^{16}O from KOH[1]	Atom%	0.18

4. Supporting Notes, Equations, Calculations

4.1 Supporting Note 1: MSCV tailing in non-porous Ni oxide layers

Figure 2a,b evidence a significant tailing in the MSCV of $^{18}O_2$ on the cathodic scan. While some previous studies indicate that slow oxygen diffusion from porous films can cause this behavior, we investigated whether tailing occurs on monolayer thick non-porous Ni films, as well. To that end, a monolayer of Ni was electrodeposited on a polished crystalline Ir disk electrode, [6] and its faradaic MSCV / CV profiles were compared to those of a polished pure Ir disk reference catalyst. Data are displayed in **Figure S12**. Indeed, even the Ni monolayer exhibited a small tailing that suggests that this delayed detection of O_2 is in part related to the charge (hole) storage mechanism and the redox processes in Ni-based OER catalysts, and not entirely due to the morphology of the Ni catalyst.

---

[1] **Contribution of ^{16}O from KOH**

Example:
in 18g water and 0.1M KOH
Number of O atoms of water are $6.02E^{+23}$
Number of O atoms of KOH are $1.07E^{+21}$

The percentage of ^{16}O atoms derived from the solid KOH is 0.18% of the total oxygen atoms in solution.
This total ^{16}O percentage from KOH water results in 0.78%.

4.2 Supporting Note 2: Faradaic oxygen efficiency (FE_{O2}) estimations

Faradic efficiencies of $Ni(OH)_2$ were derived from DEMS data obtained using the dual thin-layer flow cell setup following equation S2. The DEMS data is shown in **Figure 2**. $Q_{F,O2}^{DEMS}$ denotes the faradic charge that was exclusively used to evolve molecular oxygen (area under blue voltammogram in Figure 6). It is calculated from equation S1 from the experimental O_2 mass ion charge, $Q_{MS,j}$, (used value of $K^*_{O2,\ Ni(OH)2}$ = 8.99 10^{-6}, $K^*_{O2,\ NiFe\ LDH}$ = 8.59 10^{-6}). $Q_F^{tot}$is the experimental total anodic faradic charge comprising the charge for the generation of molecular oxygen as well as the pseudocapacitive oxidative redox charge stored in the Ni ions ($Q_{F,Ni}$), which can be expressed as the sum of the charge for the Ni^{2+} to Ni^{3+} oxidation ($Q_{F,Ni2+/3+}$) and for the Ni^{3+} to Ni^{4+} oxidation ($Q_{F,Ni3+/4+}$). The following relations hold:

$$FE_{O2} = \frac{Q_{F,O2}^{DEMS}}{Q_F^{tot}} \tag{S5}$$

$$Q_F^{tot} = Q_{F,O2}^{DEMS} + Q_{F,Ni} \tag{S6}$$

$$Q_{F,Ni} = Q_{F,Ni2+/3+} + Q_{F,Ni3+/4+} \tag{S7}$$

4.2.1 Estimation of mean FE_{O2} when integrating over peak 2 of Figure 6 (purple)

From the integration of currents in Figure 6 results the relation

$$\frac{Charge\ under\ blue\ scan\ i_{F,O2}^{DEMS}}{Anodic\ charge\ under\ peak\ 2\ of\ red\ scan\ i_F} = \frac{Q_{F,O2}^{DEMS}}{Q_{F,O2}^{DEMS}+Q_{F,Ni3+/4+}} = 0.82 \tag{S8}$$

And after rearrangement

$$0.22 \cdot Q_{F,O2}^{DEMS} = Q_{F,Ni3+/4+} \tag{S9}$$

4.2.2 Estimation of mean FE_{O2} when integrating over anodic peaks 1 and 2 of Figure 6 (pink) not including cathodic currents

From the integration of currents in Figure 6 results the relation

$$\frac{Charge\ under\ blue\ scan\ i_{F,O2}^{DEMS}}{Anodic\ charge\ under\ the\ red\ scan\ i_F} = \frac{Q_{F,O2}^{DEMS}}{Q_{F,O2}^{DEMS}+(Q_{F,Ni+2/+3}+ Q_{F,Ni+3/+4})} = 0.62 \tag{S10}$$

And after rearrangement and insertion of equation S9 follows:

$$0.39 \cdot Q_{F,O2}^{DEMS} = Q_{F,Ni+2/+3} \tag{S11}$$

From which follows using equations S9 and S11:

$$\frac{Q_{F,Ni+3/+4}}{Q_{F,Ni+2/+3}} = 0.56 \approx 0.6 \quad \text{(S12)}$$

4.3 Supporting Note 3

4.3.1 Estimation of the Oxygen mass content in Ni(OH)$_2$ and NiFe LDH catalyst

The determination of the atomic oxygen content in the Ni(OH)$_2$ and NiFe LDH catalysts is based on the analysis of the ICP-OES results. In both catalysts, the loading on GC was 0.1 mg cm^{-2} and the GC area was equal to 0.196 cm^2. This results in a total catalyst mass loading of 19.6 µg$_{tot\ cat}$. Therefore we obtain for Ni(OH)$_2$:

**Ni(OH)$_2$**	**0.1 mg cm^{-2}**	**0.196 cm^2**	**19.6 µg$_{tot\ cat}$**
**Elements/species**	**Molar Mass g/mol**		
Ni	58.69		
OH	17 · 2		
Total Ni(OH)$_2$	92.69		

$$\rightarrow \frac{19.6}{92.69} = 0.2115\ \mu mol = 211.5\ nmol_{tot\ cat}$$

Resulting total oxygen molar amount on electrode: $n_O = 423\ nmol_O$

And for NiFe LDH:

**$Ni_{0.75}Fe_{0.25}(OH)_2\ (CO_3^{2-})_{0.125} \cdot 0.5H_2O$**	**0.1 mg cm^{-2}**	**0.196 cm^2**	**19.6 µg$_{tot\ cat}$**
**Elements/species**	**Molar Mass g/mol**		
Ni	58.69 · 0.75		
Fe	55.8 · 0.25		
OH	17 · 2		
CO_3	60 · 0.125		
H_2O	18 · 0.5		
Total $Ni_{0.75}Fe_{0.25}(OH)_2\ (CO_3^{2-})_{0.125} \cdot 0.5H_2O$	108.47		

$$\rightarrow \frac{19.6}{108.47} = 0.1810\ \mu mol = 181.0\ nmol_{tot\ cat}$$

Resulting total oxygen molar amount on electrode if we consider only the oxygen in the brucite-like layers: $n_O = 362\ nmol_O$

Resulting total oxygen molar amount on electrode if we consider also the oxygen included in the intercalated water layer: $n_O = 452.5\ nmol_O$

4.3.2 Estimation of lattice oxygen participation (in %) during water oxidation

For each catalyst, a mass spectrometric ion charge, $Q_{MS,16O2}$, was calculated by the integration of the MSCV of $^{16}O_2$ of **Figure 2**. These experiments were conducted in the dual thin-layer flow cell and non-isotope labeled water was used to prepare the electrolyte. This setup is the most accurate for the absolute determination of evolved oxygen.

The DEMS mass ion charge $Q_{MS,16O2}$ represents the molecular oxygen produced over the cyclic voltammetric scan. To obtain the fractional % of oxygen charge associated with the LOER mechanism, $Q_{MS,LOER}$, we now multiply $Q_{MS,16O2}$ with λ / (1+λ), where λ= $Q_{MS,16O18O}/\ Q_{MS,18O18O}$ is the ratio of isotope mass ion charges taken from the isotope experiment in **Figure 3e**. The values of λ from Figure 3e used here are 0.1 and 0.17 for NiFe LDH and $Ni(OH)_2$, respectively. Finally, the DEMS-derived faradaic charge, $Q_{F,LOER}^{DEMS}$ is obtained after dividing $Q_{MS,LOER}$ by the electron-normalized calibration constant K*/n (K* ($K^*_{O2,\ Ni(OH)2}$ = 8.99 10^{-6}, $K^*_{O2,\ NiFe\ LDH}$ = 8.59 10^{-6}, n=4). After dividing $Q_{F,LOER}^{DEMS}$ by 4F one obtains the molar amount of O_2 evolved via the LOER mechanism.

NiFe LDH	***Ni(OH)₂***
$Q_{MS,16O2} = 4.9 \cdot 10^{-8}\mathrm{C}$<br>$Q_{MS,LOER} = 4.9 \cdot 10^{-8}C \cdot 0.09 = 4.41 \cdot 10^{-9}C$<br>$n_{LOER}^{O2} = \frac{Q_{F,LOER}^{DEMS}}{4 \cdot F} = \frac{Q_{MS,LOER} \cdot 4}{K^* \cdot 4 \cdot F} = 5.32 \cdot 10^{-9}\ mol_{O2}$<br>Therefore, compared to ICP-OES:<br>$\frac{2 \cdot 5.32 \cdot 10^{-9}\ mol_O}{362 \cdot 10^{-9}\ mol_O} = 0.029$<br>$\sim \mathbf{2.9}$ %	$Q_{MS,16O2} = 4.7 \cdot 10^{-8}\ \mathrm{C}$<br>$Q_{MS,LOER} = 4.7 \cdot 10^{-8}C \cdot 0.14 = 6.58 \cdot 10^{-9}\ \mathrm{C}$<br>$n_{LOER}^{O2} = \frac{Q_{F,LOER}^{DEMS}}{4 \cdot F} = \frac{Q_{MS,LOER} \cdot 4}{K^* \cdot 4 \cdot F} = 7.58 \cdot 10^{-9}\ mol_{O2}$<br>Therefore, compared to ICP-OES:<br>$\frac{2 \cdot 7.58 \cdot 10^{-9}\ mol_O}{423 \cdot 10^{-9}\ mol_O} = 0.036$<br>$\sim \mathbf{3.6}$ %

4.3.3 The molar fraction of redox-active Ni ions from voltammetric $Ni^{2+/3+}$ redox wave

Analysis made from the electrochemical measurement with the dual thin-layer flow cell setup on NiFe LDH and $Ni(OH)_2$. Based on the integration of the anodic Ni oxidation peak in Figure 2, the redox active Ni amount, $n_{Ni,redox}$, is :

$$For\ NiFe\ LDH\colon n_{Ni,redox} = 2.5 \cdot 10^{-9} mol = 0.25 \cdot 10^{-2} \mu mol$$

$$For\ Ni(OH)_2\colon n_{Ni,redox} = 3.9 \cdot 10^{-7} mol = 0.39 \mu mol$$

From ICP-OES the weight percentage of Ni atoms in the catalyst is known:

	**Catalyst load (mg cm^{-2})**	**Ni (wt%)**	**Fe (wt%)**	**Total metal (wt%)**	**Total metal (mg cm^{-2})**
NiFe LDH	0.1	43.41	10.17	53.58	0.05358
$Ni(OH)_2$	0.1	78.45		78.45	0.07845

Therefore, the molar fraction of redox-active Ni ions relative to the total Ni content can be calculated:

NiFe LDH	***Ni(OH)₂***
$m_{Ni} = 0.1 \frac{mg}{cm^2} \cdot 0.196 cm^2 \cdot \frac{43.41}{100} = 0.008508 mg_{Ni} = 8.51 \mu g_{Ni}$ $n_{Ni} = \frac{m_{Ni}}{M_{Ni}} = \frac{8.51 \mu g_{Ni}}{58.693\, g/mol} = 0.145 \mu mol_{Ni}$ $\frac{n_{Ni,redox}}{n_{Ni}} = \frac{0.25 \cdot 10^{-2} \mu mol_{Ni}}{0.145\ \mu mol_{Ni}} = 01.72 \times 10^{-2} = \mathbf{1.72\%}$	$m_{Ni} = 0.1 \frac{mg}{cm^2} \cdot 0.196 cm^2 \cdot \frac{78.45}{100} = 0.01538 mg_{Ni} = 15.4 \mu g_{Ni}$ $n_{Ni} = \frac{m_{Ni}}{M_{Ni}} = \frac{15.4 \mu g_{Ni}}{58.693\, g/mol} = 0.2624 \mu mol_{Ni}$ $\frac{n_{Ni,redox}}{n_{Ni}} = \frac{0.39\ \mu mol_{Ni}}{0.2624\ \mu mol_{Ni}} = 1.48 = 148\% > 100\%$

4.4 Supporting Note 4

Details of the calculation for mass activity - Highest metal mass activity reached in the DEMS scan.

For both catalysts the highest current density reached was 3.7 mA cm^{-2} for NiFe LDH and 3.1 mA cm^{-2} for $Ni(OH)_2$ using the hanging droplet flow cell setup in electrolyte solution of 0.1 M KOH (99.3% labelled water) at a scan rate 10 mV s^{-1}. The total metal based mass activity (MA) reached in the DEMS scan can then be calculated:

In *NiFe LDH*

$$MA = 3.7 \cdot \frac{mA}{cm^2} \cdot \frac{1}{0.05358} \cdot \frac{cm^2}{mg} = 69.06 \sim 70 \cdot \frac{mA}{mg} \sim 0.07 \cdot \frac{A}{mg_{metal}}$$

And in *Ni(OH)₂*

$$MA = 3.1 \cdot \frac{mA}{cm^2} \cdot \frac{1}{0.07845} \cdot \frac{cm^2}{mg} = 39.52 \sim 40 \cdot \frac{mA}{mg} \sim 0.04 \cdot \frac{A}{mg_{metal}}$$

We compared the mass activity with the value reported in ref. [7], which presents a NiFe catalyst with mass activity of $\sim 0.4 \frac{A}{mg_{metal}}$. This calculation reveals a significant low mass activity during our experiments, which possibly allows us for an easier observation of the LOER process for a long period of time.

5. References

[1] F. Dionigi, T. Reier, Z. Pawolek, M. Gliech, P. Strasser, *Chemsuschem* **2016**, *9*, 962-972.

[2] L. Trotochaud, S. L. Young, J. K. Ranney, S. W. Boettcher, *Journal of the American Chemical Society* **2014**, *136*, 6744-6753.

[3] O. Wolter, J. Heitbaum, *Berichte der Bunsengesellschaft für physikalische Chemie* **1984**, *88*, 2-6.

[4] H. Baltruschat, *Journal of the American Society for Mass Spectrometry* **2004**, *15*, 1693-1706.

[5] Z. Jusys, H. Massong, H. Baltruschat, *J Electrochem Soc* **1999**, *146*, 1093-1098.

[6] E. Özer, I. Sinev, A. Mingers, J. Araujo, T. Kropp, M. Mavrikakis, K. Mayrhofer, B. Cuenya, P. Strasser, *Surfaces* **2018**, *1*, 165-186.

[7] C. Roy, B. Sebok, S. B. Scott, E. M. Fiordaliso, J. E. Sorensen, A. Bodin, D. B. Trimarco, C. D. Damsgaard, P. C. K. Vesborg, O. Hansen, I. E. L. Stephens, J. Kibsgaard, I. Chorkendorff, *Nat Catal* **2018**, *1*, 820-829.
